# Supplementary material for: Community support for injured patients: A scoping review and narrative synthesis
Source: PLoS One. 2024 Feb 1;19(2):e0289861. doi: 10.1371/journal.pone.0289861 (PMC10833531; doi:10.1371/journal.pone.0289861)
Supplement: S1 File — (DOCX) [file pone.0289861.s002.docx]

**SUPPLEMENT 1: SEARCH STRATEGIES BY DATABASE**

PubMed / Medline (National Library of Medicine, NCBI)

2,343 records March 1, 2023

("Self-Help Groups"[Mesh:NoExp] OR community engagement[tiab] OR community group*[tiab] OR community support[tiab] OR help group*[tiab] OR patient support[tiab] OR peer support[tiab] OR peer counsel*[tiab] OR support group*[tiab] OR online support[tiab])

AND

("Wounds and Injuries"[Mesh] OR "Accidents"[Mesh] OR "Violence"[Mesh] OR accidental[tiab] OR accidents[tiab] OR burn[tiab] OR burns[tiab] OR domestic abuse[tiab] OR fallers[tiab] OR falls[tiab] OR fractures[tiab] OR gunshot[tiab] OR injuries[tiab] OR injury[tiab] OR injured[tiab] OR partner abuse[tiab] OR physical abuse[tiab] OR spousal abuse[tiab] OR spouse abuse[tiab] OR stabbing*[tiab] OR trauma[tiab] OR traumas[tiab] OR wound[tiab] OR wounds[tiab] OR violence[tiab])

Cumulative Index to Nursing and Allied Health Literature (CINAHL Complete, EBSCOhost)

1077 records, March 1, 2023

Advanced Search

Search Options

remove:

Apply equivalent subjects

select:

Publication Type: Journal Article

MH "Support Groups" OR MH "Peer Counseling"

OR title OR abstract:

"community engagement" OR "community group*" OR "community support" OR "help group*" OR "patient support" OR "peer support" OR "support group*" OR "online support"

AND

MH "Wounds and Injuries+" OR MH "Accidents+" OR MH "Trauma" OR MH "Violence+"

OR title OR abstract

accidental OR accidents OR burn OR burns OR "domestic abuse" OR fallers OR falls OR fractures OR gunshot OR injuries OR injury OR injured OR "partner abuse" OR "physical abuse" OR "spousal abuse" OR "spouse abuse" OR stabbing* OR trauma OR traumas OR wound OR wounds OR violence

Web of Science Core Collection (Clarivate)

1,569 records, March 1, 2023

Editions:

Science Citation Index Expanded

Social Sciences Citation Index

Advanced Search

More Options: Exact Search

#1

(TI=("community engagement" OR "community group*" OR "community support" OR "help group*" OR "patient support" OR "peer support" OR "support group*" OR "online support")) OR AB=("community engagement" OR "community group*" OR "community support" OR "help group*" OR "patient support" OR "peer support" OR "support group*" OR "online support")

AND

(TI=(accidental OR accidents OR burn OR burns OR domestic abuse OR fallers OR falls OR fractures OR gunshot OR injuries OR injury OR injured OR "partner abuse" OR "physical abuse" OR "spousal abuse" OR "spouse abuse" OR stabbing* OR trauma OR traumas OR wound OR wounds OR violence)) OR (AB=( accidental OR accidents OR burn OR burns OR domestic abuse OR fallers OR falls OR fractures OR gunshot OR injuries OR injury OR injured OR "partner abuse" OR "physical abuse" OR "spousal abuse" OR "spouse abuse" OR stabbing* OR trauma OR traumas OR wound OR wounds OR violence))

#1 AND #2

PubMed: 2,343

CINAHL: 1077

Web of Science Core Collection: 1,569

total # of references retrieved across 3 databases March 1, 2023: 4,989
