# Supplementary material for: Community support for injured patients: A scoping review and narrative synthesis
Source: PLoS One. 2024 Feb 1;19(2):e0289861. doi: 10.1371/journal.pone.0289861 (PMC10833531; doi:10.1371/journal.pone.0289861)
Supplement: Attachment — Submitted filename: PONE-D-23-23344_HA1.1.pdf [file pone.0289861.s005.pdf]

PLOS ONE

Community Support for Injured Patients: A Scoping Review and Narrative Synthesis

--Manuscript Draft--

**Manuscript Number:**	PONE-D-23-23344R1
**Article Type:**	Research Article
**Full Title:**	Community Support for Injured Patients: A Scoping Review and Narrative Synthesis
**Short Title:**	Community Support Injured Patients
**Corresponding Author:**	Rashi Jhunjhunwala<br>Beth Israel Deaconess Medical Center<br>Boston, MA UNITED STATES
**Keywords:**	community support; Injury; trauma; support groups; Peer Support
**Abstract:**	BACKGROUND: Community-based peer support (CBPS) groups have been effective in facilitating access to and retention in the healthcare system for patients with HIV/AIDS, cancer, diabetes, and other communicable and non-communicable diseases. Given the high incidence of morbidity that results from traumatic injuries, and the barriers to reaching and accessing care for injured patients, community-based support groups may prove to be similarly effective in this population. OBJECTIVES: The objective of this review is to identify the extent and impact of CBPS for injured patients. ELIGIBILITY: We included primary research on studies that evaluated peer-support groups that were solely based in the community. Hospital-based or healthcare-professional led groups were excluded. EVIDENCE: Sources were identified from a systematic search of Medline / PubMed, CINAHL, and Web of Science Core Collection. CHARTING METHODS: We utilized a narrative synthesis approach to data analysis. RESULTS: 4,989 references were retrieved; 25 were included in final data extraction. There was a variety of methodologies represented and the groups included patients with spinal cord injury (N=2), traumatic brain or head injury (N=7), burns (N=4), intimate partner violence (IPV) (N=5), mixed injuries (N=5), torture (N=1), and brachial plexus injury (N=1). Multiple benefits were reported by support group participants; categorized as social, emotional, logistical, or educational benefits. CONCLUSIONS: community-based peer support groups can provide education, community, and may have implications for retention in care for injured patients.
**Order of Authors:**	Rashi Jhunjhunwala, MD MA
	Anusha Jayaram, MD, MBA
	Carol Mita, MLIS
	Justine Davies, MD
	Kathryn Chu, MD MPH FACS FASCRS
**Response to Reviewers:**	Dear Reviewers and Academic Editor,<br><br>Thank you very much for your thorough evaluation of our manuscript, “Community Support for Injured Patients: A Scoping Review and Narrative Synthesis.” We have responded to the comments and suggestions made by all reviewers, and edited the manuscript as suggested. Please see below for our point-by-point responses. Both a marked/track changes version and unmarked version of the revised manuscript have also been submitted for review.<br><br>Best,<br>Rashi Jhunjhunwala and co-authors.<br><br>Reviewer #1<br>COMMENT: Although the topic of study is very important, the article is not suitable for publication.<br>RESPONSE: We hope you will change this opinion after reviewing our revised manuscript.

COMMENT: Range of time interval of studies under review is very wide.
RESPONSE: We do not view this as a detriment to our study. We aimed to evaluate the research that has been done on community-based peer support groups for injured patients, and we wanted to include all research that we were able to access. We did not see a need to limit our search to any specific time frame.

COMMENT: Table 1 could have been presented in prose, Table 2 should have been attached to the article.
RESPONSE: We chose to present table 1 in table format because we found it easier to quickly reference inclusion/exclusion criteria in this manner. Additionally, this is a format that is commonly used in scoping reviews. Table 2 is included at the bottom of the manuscript and was able to be located by other reviewers.

COMMENT: Poor graphs are presented, no statistical methods are used to answer the researcher's question, and it does not add new findings to the existing literature.
RESPONSE: The methodology utilized in this review is detailed in the Methods section of the manuscript. While no statistical methods were included, there are other valid research methodologies that exist; one of these is narrative synthesis. We refer the reviewer to the following publication from the Joanna Briggs Institute to gain insight into the ways data can be analyzed and presented in a scoping review: Pollock, Danielle1; Peters, Micah D.J.2,3,4; Khalil, Hanan5; McInerney, Patricia6; Alexander, Lyndsay7,8; Tricco, Andrea C.9,10,11; Evans, Catrin12; de Moraes, Érica Brandão13,14; Godfrey, Christina M.11; Pieper, Dawid15,16; Saran, Ashrita17,18; Stern, Cindy1; Munn, Zachary1. Recommendations for the extraction, analysis, and presentation of results in scoping reviews. JBI Evidence Synthesis 21(3):p 520-532, March 2023. | DOI: 10.11124/JBIES-22-00123

Reviewer #2

COMMENT: The studies in this review identified positive benefits of community support groups for injured people, including creation of a group identity and regaining of self-esteem, emotional support and guidance, and delivery of practical knowledge about their disease, recovery and treatment process, or ways to access medical, legal, or social support. However, there are inconsistencies in the fonts, some of which are neo-Roman. Please correct it as requested by PLOS ONE.
RESPONSE: Fonts have been standardized to adhere to PLOS ONE guidelines.

Reviewer #3

COMMENT: The aim of the review appears broader than what is initially stated in the research question. Clarifying the alignment between the research question and the review's objectives would enhance the paper's coherence.
RESPONSE: Thank you for this comment. We have revised the aims and research question.
The aims now read: “This scoping review aims to identify the extent, distribution, benefits, utility, and impact of community-based peer support groups for injured patients”.
The research question reads: “what research has been done on the extent, distribution, benefits, utility, and impact of health-system independent community-based support groups (i.e. those which are not based in healthcare facilities or run by healthcare professionals) for injured persons?”

COMMENT: It's noted that 13 studies were excluded due to unavailability of full texts, but there is no mention of efforts made to retrieve them, such as contacting the authors. Additionally, the potential impact of excluding these studies on the conclusions should be addressed in the limitations section.
RESPONSE: This is a very valid point. Many of the results that we were unable to find were find were from the 1980s and early 1990s and contact information for the authors were not available. We have given more detail regarding the extent to which attempts were made to retrieve the articles in the first section of the “Results” – lines 180-183. The text now reads:
“Full text was not recoverable for 13 studies due to unavailability online through Harvard libraries, inter-library loan, or other formal retrieval mechanisms. Four of 13

	unavailable studies were published prior to 1990 and contact information for the authors was not available. Attempts to contact the other 9 authors were unsuccessful." We have also included the fact that these were not retrieved in the limitations, as suggested (lines 448-449): There were 13 (out of 112) articles not found by our search strategy that could have added additional insight.<br><br>COMMENT: The discussion section requires a complete rewrite rather than just improvements. It lacks a clear flow and connection with the results section. Instead, it introduces new information that should have been integrated into the results. Furthermore, the emphasis on the scarcity of studies from low and middle-income countries diverts attention from discussing the actual results and comparing them with other studies.<br>RESPONSE: The discussion has been re-structured to first state main results, discussion of those results, the context in the literature with other conditions, the unique challenges of injury/trauma in formthe lack of literature in LMICs, limitations, and conclusion. The discussion on LMICs and socioeconomic element has been paired down to discuss it as an important context to injury burden, but not to take away from the main results.<br><br>COMMENT: I have attached a file with more detailed comments for your reference.<br>RESPONSE: Thank you for your thorough and thoughtful comments. We have aimed to respond to them in the track-changes document we have attached for review. These include:<br>-We have standardized the language regarding "community-based peer support groups" throughout the document, and included an acronym, CBPR.<br>-Revised aims and research question, as stated above.<br>-Included more information about missing studies, as stated above.<br>-Included references for each component of the "study characteristics" section of the Results.<br>-Defined each category of "social, emotional, educational, logistical" benefits immediately prior to "figure 2".<br>-Revised/rewrote discussion, as stated above.<br>-Various sentences/wording have been revised as recommended.
**Additional Information:**	
**Question**	**Response**
**Financial Disclosure**<br><br>Enter a financial disclosure statement that describes the sources of funding for the work included in this submission. Review the submission guidelines for detailed requirements. View published research articles from *PLOS ONE* for specific examples.<br><br>This statement is required for submission and **will appear in the published article** if the submission is accepted. Please make sure it is accurate.	This study was conducted as part of the Equi-Injury Study, funded by the UK National Institute of Health and Care Research, NIHR Global Health Groups Call 3, application 133135. Authors who received award are JD and KC. The funders had no role in study design, data collection and analysis, decision to publish, or preparation of the manuscript.

**Unfunded studies**<br>Enter: *The author(s) received no specific funding for this work.*<br><br>**Funded studies**<br>Enter a statement with the following details:<br>• Initials of the authors who received each award<br>• Grant numbers awarded to each author<br>• The full name of each funder<br>• URL of each funder website<br>• Did the sponsors or funders play any role in the study design, data collection and analysis, decision to publish, or preparation of the manuscript?<br>• **NO** - Include this sentence at the end of your statement: *The funders had no role in study design, data collection and analysis, decision to publish, or preparation of the manuscript.*<br>• **YES** - Specify the role(s) played.<br><br>* typeset	
**Competing Interests**<br><br>Use the instructions below to enter a competing interest statement for this submission. On behalf of all authors, disclose any competing interests that could be perceived to bias this work—acknowledging all financial support and any other relevant financial or non-financial competing interests.<br><br>This statement is required for submission and **will appear in the published article** if the submission is accepted. Please make sure it is accurate and that any funding sources listed in your Funding Information later in the submission form are also declared in your Financial Disclosure statement.<br><br>View published research articles from *PLOS ONE* for specific examples.	The authors have declared that no competing interests exist.

**NO authors have competing interests**<br><br>Enter: *The authors have declared that no competing interests exist.*<br><br>**Authors with competing interests**<br><br>Enter competing interest details beginning with this statement:<br><br>*I have read the journal's policy and the authors of this manuscript have the following competing interests: [insert competing interests here]*<br><br>* typeset	
**Ethics Statement**<br><br>Enter an ethics statement for this submission. This statement is required if the study involved:<br><br>• Human participants<br>• Human specimens or tissue<br>• Vertebrate animals or cephalopods<br>• Vertebrate embryos or tissues<br>• Field research<br><br>Write "N/A" if the submission does not require an ethics statement.<br><br>General guidance is provided below. Consult the submission guidelines for detailed instructions. **Make sure that all information entered here is included in the Methods section of the manuscript.**	N/A

Powered by Editorial Manager® and ProduXion Manager® from Aries Systems Corporation

**Format for specific study types**<br><br>**Human Subject Research (involving human participants and/or tissue)**<br>• Give the name of the institutional review board or ethics committee that approved the study<br>• Include the approval number and/or a statement indicating approval of this research<br>• Indicate the form of consent obtained (written/oral) or the reason that consent was not obtained (e.g. the data were analyzed anonymously)<br><br>**Animal Research (involving vertebrate animals, embryos or tissues)**<br>• Provide the name of the Institutional Animal Care and Use Committee (IACUC) or other relevant ethics board that reviewed the study protocol, and indicate whether they approved this research or granted a formal waiver of ethical approval<br>• Include an approval number if one was obtained<br>• If the study involved *non-human primates*, add *additional details* about animal welfare and steps taken to ameliorate suffering<br>• If anesthesia, euthanasia, or any kind of animal sacrifice is part of the study, include briefly which substances and/or methods were applied<br><br>**Field Research**<br><br>Include the following details if this study involves the collection of plant, animal, or other materials from a natural setting:<br>• Field permit number<br>• Name of the institution or relevant body that granted permission	
**Data Availability**<br><br>Authors are required to make all data underlying the findings described fully available, without restriction, and from the time of publication. PLOS allows rare exceptions to address legal and ethical concerns. See the PLOS Data Policy and FAQ for detailed information.	Yes - all data are fully available without restriction

A Data Availability Statement describing where the data can be found is required at submission. Your answers to this question constitute the Data Availability Statement and **will be published in the article**, if accepted.<br><br>**Important:** Stating 'data available on request from the author' is not sufficient. If your data are only available upon request, select 'No' for the first question and explain your exceptional situation in the text box.<br><br>Do the authors confirm that all data underlying the findings described in their manuscript are fully available without restriction?	
**Describe where the data may be found in full sentences. If you are copying our sample text, replace any instances of XXX with the appropriate details.**<br><br>• If the data are **held or will be held in a public repository**, include URLs, accession numbers or DOIs. If this information will only be available after acceptance, indicate this by ticking the box below. For example: *All XXX files are available from the XXX database (accession number(s) XXX, XXX.*).<br>• If the data are all contained **within the manuscript and/or Supporting Information files**, enter the following: *All relevant data are within the manuscript and its Supporting Information files.*<br>• If neither of these applies but you are able to provide **details of access elsewhere**, with or without limitations, please do so. For example:<br><br>*Data cannot be shared publicly because of [XXX]. Data are available from the XXX Institutional Data Access / Ethics Committee (contact via XXX) for researchers who meet the criteria for access to confidential data.*<br><br>*The data underlying the results presented in the study are available from (include the name of the third party*	All relevant data are within the manuscript and its Supporting Information files.

and contact information or URL).<br>• This text is appropriate if the data are owned by a third party and authors do not have permission to share the data.<br><br>* typeset	
Additional data availability information:	

Manuscript

Click here to access/download;Manuscript;Community Support for Injured Patients_REVISION_Final_Clean.docx

Title: Community Support for Injured Patients: A Scoping Review and Narrative Synthesis

Authors:
Rashi Jhunjhunwala MD MA,[1,2] Anusha Jayaram MD MBA, [1,2] Carol Mita MLIS,[3] Justine Davies MD*[4,5], Kathryn Chu MD MPH FACS FASCRS*[5]
* Joint last authorship

Affiliations:
1. Program in Global Surgery and Social Change, Harvard Medical School, Boston, Massachusetts, USA
2. Department of Surgery, Beth Israel Deaconess Medical Center, Boston, Massachusetts, USA
3. Countway Library, Harvard Medical School, Boston, Massachusetts, USA
4. Institute of Applied Health Research, University of Birmingham, Birmingham, UK
5. Centre for Global Surgery, Department of Global Health, Faculty of Medicine and Health Sciences, Stellenbosch University, Stellenbosch, South Africa

Corresponding Author:
Prof Justine Davies; J.Davies.6@bham.ac.uk

Corresponding Author for Submission:
Rashi Jhunjhunwala; rjhunjhu@bidmc.harvard.edu

Word Count: 3452

Abstract:

BACKGROUND: Community-based peer support (CBPS) groups have been effective in facilitating access to and retention in the healthcare system for patients with HIV/AIDS, cancer, diabetes, and other communicable and non-communicable diseases. Given the high incidence of morbidity that results from traumatic injuries, and the barriers to reaching and accessing care for injured patients, community-based support groups may prove to be similarly effective in this population. OBJECTIVES: The objective of this review is to identify the extent and impact of CBPS for injured patients. ELIGIBILITY: We included primary research on studies that evaluated peer-support groups that were solely based in the community. Hospital-based or healthcare-professional led groups were excluded. EVIDENCE: Sources were identified from a systematic search of Medline / PubMed, CINAHL, and Web of Science Core Collection. CHARTING METHODS: We utilized a narrative synthesis approach to data analysis. RESULTS: 4,989 references were retrieved; 25 were included in final data extraction. There was a variety of methodologies represented and the groups included patients with spinal cord injury (N=2), traumatic brain or head injury (N=7), burns (N=4), intimate partner violence (IPV) (N=5), mixed injuries (N=5), torture (N=1), and brachial plexus injury (N=1). Multiple benefits were reported by support group participants; categorized as social, emotional, logistical, or educational benefits. CONCLUSIONS: community-based peer support groups can provide education, community, and may have implications for retention in care for injured patients.

Introduction:

The World Health Organization (WHO) estimates that 4.4 million people die from injuries such as acts of violence, road traffic accidents, falls, and burns annually, and 90% of these deaths occur in low- and middle-income countries (LMICs) (1). Millions more suffer non-fatal injuries that require extensive medical care and support (2). Injured patients often face many barriers to seeking, reaching, and receiving care (3). These barriers can prevent or delay rehabilitation that allows for return to optimal function, especially after the acute injury has been addressed. Given the potentially chronic conditions that result from injury, retention in care is necessary. However, injured peoples' needs go beyond retention in medical care; they can also require care for ongoing psychological issues or matters of daily living which are often not addressed within healthcare systems.

For patients with other medical conditions, peer and community support has been shown to facilitate access to healthcare through improved retention of current care, introduction to other providers of care, and provision of information and psychological support (4–7). Peer support has long been used within mental health services and has been defined as social emotional support that is mutually offered or provided by those with similar lived experiences (8). This process of support, companionship, and assistance often counter feelings of loneliness, discrimination, and frustration. The most common form of peer support has been self-help groups, which have been defined as a voluntary small group for mutual aid (9). Studies on peer support amongst HIV patients also demonstrate increased retention in care, improved anti-retroviral therapy adherence and viral suppression, and increased financial and moral support (10,11). Further, studies of pregnant women demonstrate the positive impact that peer-support can have on motherhood and coping

with issues like substance use (12,13). Engaging the community, especially for historically marginalized groups, has also been demonstrated to result in positive health outcomes (14,15). Further, meaningful engagement of and advocacy by members of the HIV/AIDS community has been shown to lead to improved HIV/AIDS services and policy changes that lead to better access to care and service provision (16,17).

Despite the similar needs of injured patients for ongoing medical care and the often life-changing nature of their injuries requiring psychological and physical support, there is little known about whether community-based peer support (CBPS) groups exist, benefit injured persons, or whether they play a role improving and retaining access to acute and chronic injury care. This scoping review aims to identify the extent, distribution, benefits, utility, and impact of community-based peer support groups for injured patients.

Methods:

This scoping review is conducted based on the expanded Arksey and O'Malley framework and uses PRISMA-ScR guidelines (18,19).

Defining the Research Question:

We aimed to answer the question: what research has been done on the extent, distribution, benefits, utility, and impact of health-system independent community-based support groups (i.e. those which are not based in healthcare facilities or run by healthcare professionals) for injured persons?

Search Strategy:

Studies reporting on CBPS groups for physical injuries were identified by a systematic search of Medline / PubMed (National Library of Medicine, NCBI); CINAHL (CINAHL Complete, EBSCOhost), and Web of Science Core Collection (Clarivate). Controlled vocabulary terms (i.e., MeSH; CINAHL thesaurus subject headings) were included when available and appropriate. The search strategies were designed and carried out by a librarian (CM). No language limits or date restrictions were applied. The exact search terms used for each of the databases are provided in the supplementary document.

These searches were undertaken on March 1st, 2023. We also searched the grey literature via Google searches, reviewed injury society and trauma society web pages, and communicated with global experts in the field of injury research to identify additional studies evaluating the impact of these groups.

Study Inclusion and Exclusion:

Study inclusion and exclusion criteria are shown in **Table 1**. We included primary research studies as well as those which describe advocacy or policy changes to manage injuries, since community support groups have been advocates for improved care for other conditions such as HIV/AIDS (16,17).

We excluded studies that evaluated groups that were hospital or rehabilitation facility-based or led/created by healthcare providers. We also excluded all studies that focused on Post Traumatic Stress Disorder (PTSD) where it was not clear that the PTSD occurred because of physical injury.

Table 1. Inclusion and exclusion criteria

Inclusion	Exclusion
• All publications through March 1, 2023<br>• Studies of community or patient groups focused on peer-support of people who have been injured and evaluated the impact of community or patient groups for those people<br>• Studies that assess the needs and priorities of community-based patient groups for people who have been injured<br>• All countries<br>• English language	• Studies that evaluated groups that were either led or created by hospital-affiliated staff, healthcare providers, or rehabilitation facilities<br>• Studies of community support groups that focused on injury prevention, self-harm, health issues not related to the injury, or groups that focused only on mental health or Post Traumatic Stress Disorder<br>• Support groups that did not specify that the participants had experienced physical injury

Study Screening & Data Extraction

All studies found were uploaded into Covidence for screening and data extraction. Covidence is a web-based collaboration software platform that streamlines the production of systematic and other literature reviews.(20) Two reviewers (RJ, AJ) screened all titles and abstracts independently for inclusion. In case of disagreement, RJ and AJ resolved conflicts through discussion. This process was repeated for full text review in Covidence.

A data extraction form was developed and each of two reviewers independently extracted data from each article, after which consensus was reached through review and discussion. Study data points collected were study design, funding, injury type, the study population, number of participants, what content was shared in the group, and discussion themes in the group. We also collected data on the country in which the support group was based, the country of the study's first and last author, and the years the study was conducted and published.

Data Analysis

We approached the data analysis for this review via a narrative synthesis methodology. This methodology was chosen as it is a widely utilized and accepted mechanism for conducting scoping reviews, as it is a way to synthesize and aggregate the existing body of knowledge on a topic to then elucidate areas of opportunity for further investigation. Since our inclusion criteria did not preclude any specific study methodologies, we expected variety and heterogeneity in our final study sample. We did not assess study quality in this scoping review as we believed all relevant articles per the inclusion criteria should be assessed.

Funding

This study was funded in part by the Equi-Injury Study, funded by the UK National Institute of Health and Care Research, NIHR Global Health Groups Call 3, application 133135.

Synthesis of Results

The outcomes were grouped into three categories. The first category was study characteristics, which includes study methodology, years during which the studies were conducted, geographic location of support groups, and presence or absence of study funding. We next reported on support group characteristics, including type of injury, number of participants, leadership, and facilitation of the groups, and whether the group itself received funding. Finally, we reported the summary of benefits described by the support group members – this was categorised by outcomes for all injury types together and then by specific injury type.

Results:

Study Characteristics

4,989 references (PubMed: 2343; CINAHL: 1077; Web of Science: 1569) were retrieved from the database searches on March 1, 2023. Duplicate records were removed using EndNote; import into Covidence resulted in 3489 unique references for screening. Of these, 112 were included for full text review. Full text was not recoverable for 13 studies due to unavailability online through Harvard libraries, inter-library loan, or other formal retrieval mechanisms. Four of 13 unavailable studies were published prior to 1990 and contact information for the authors was not available. Attempts to contact the other nine authors were unsuccessful. Of the 99 full texts that were reviewed, 25 studies met criteria for data extraction (**Figure 1).**

Figure 1. PRISMA Flow diagram

Study characteristics

Of the 25 studies included, eleven used purely qualitative methodology (23, 24, 26- 28, 31, 33- 35, 37, 42), six were cross-sectional quantitative studies (22, 29, 30, 36, 40, 45), three used mixed-methods (41, 43, 44), three were case reports (25, 38, 39), and there were two randomized controlled trials (21, 32). Studies were published from 1981 to 2022. Eleven studies were conducted in the US (21, 26, 28, 30, 37- 42, 44), seven in Canada (23, 24, 32- 35, 39), and three in both countries (22, 36, 43). There was one study each from the UK (45) and South Africa (25). There were two studies in which the peer support occurred in the form of online forums and thereby were not limited by geographic region (27, 31). **Table 2** presents an overview of the 25 studies included in data extraction.

Support Group Characteristics

CBPS groups have been studied for persons with spinal cord injury (N=2), traumatic brain or head injury (N=7), burns (N=4), intimate partner violence (IPV) (N=5), mixed injuries (N=5), torture (N=1), and brachial plexus injury (N=1)**.** The number of support group members ranged from 2 to 60+, with most of the groups reporting 20-40 active members. Fifteen studies reported on whether group leadership was provided by members themselves or an external facilitator such as professional facilitators, therapists, social workers, or social work or therapy students (21, 24, 25, 28, 30, 32-34, 38-42, 44, 45). Of these fifteen studies, seven (24, 28, 38, 40, 41, 44, 45) reported that the groups were run by professionals rather than participant volunteers, two groups were run by non-professional facilitators (30, 42). Five groups reported that peers volunteered or rotated through leading the groups (21, 25, 32, 34, 39) and one group utilized one-to-one peer mentorship (33). Data about the group facilitators were unavailable or unclear for the remaining studies. Seven peer-support groups reported receiving funding for the groups' activities or meetings – one from the Alberta Worker's Compensation Board (29) and six from philanthropic organizations (26, 28, 34, 41, 43, 44).

Benefits Reported by Support Group Participants

There were a variety of positive benefits reported by support group members. These were grouped into educational benefits, social benefits, emotional benefits, and logistical benefits **(Figure 2)**. Educational benefits include explanations and descriptions of various disease processes and what participants could expect for their disease course. Social benefits were defined as those benefits that ameliorated the participants' social network, solidarity with

others with similar lived experiences, and community building. Emotional benefits are those benefits that participants indicated were impactful to their sense of self or emotional safety and wellbeing. Lastly, logistical benefits are those that directly pertain to making connections with aid services, legal assistance, guidance on workers' compensation claims, and other administrative issues.

Figure 2. Categories of peer support reported in all studies; number of studies per grouping indicated within boxes.

Thirteen of 25 studies concluded that shared educational or didactic material in the group was a benefit of participation (21–33). Eight studies reported that they valued and appreciated the sharing of medical knowledge, either via dissemination from the group leadership or between participants within the groups (24,26–30,32,34).

From a social perspective, nineteen studies reported that support group members indicated they were able to obtain guidance and coping strategies as a result of group participation (21,23–26,28–33,35–42), and fourteen studies reported that the groups created a sense of solidarity and group identity that members found lacking in other areas of their lives (24,26,30,33–35,37–39,41–44,25). Social interaction was mentioned as a positive outcome in eleven studies (22–24,26,32,33,36,38,40,42,44).

Emotional outcomes constituted a substantial portion of the reported benefits. Fourteen of the studies reported that participants found emotional support and/or catharsis as a function of group membership (21,24,26–29,31–33,38–42), and fourteen studies reported that group members felt a sense of not feeling alone (22–24,28,29,31,33–35,37,39,40,42,44) after they joined the group. Group participants in eleven studies

reported a benefit to their self-esteem, empowerment, and overall quality of life (22,24,25,32,34,35,37,38,40–42). Six studies noted that support groups gave participants a sense of hope for the future (26,32,35,37,39,42). Finally, seven studies reported that participants felt a sense of altruism and being able to give back to other injury survivors as a result of group participation (25,26,28,30,35,37,39). On a logistical level, eleven studies reported that group members found that they were able to gain assistance in navigating the health, legal, and/or social systems from their peers (23,26–30,32,40–42,45).

We also evaluated the types of benefits reported by groups for people with specific types of injuries. We focused on studies reporting on brain injury support groups, burn injury support groups, intimate partner violence (IPV) support groups, and mixed injury support groups, as these injury types were the subject of more than two studies included in this review. Table 3 shows the distribution of each type of support reported by each injury group.

Table 3. Peer support type reported by injury group

**Injury type**	Total # of Groups	Emotional support	Educational support	Social support	Logistical support
Brain injury	7	7	7	7	4
Burn injury	4	3	1	4	0
IPV	5	4	1	5	2
Mixed injury	5	4	2	4	4

Considering the types of benefits reported by people with specific types of injuries, out of the seven studies reporting on brain injury support groups, six studies mentioned that participants guidance and coping strategies from the groups (21,26,28,30,32,33). Of the four studies reporting on persons with burn injuries, three reported that participants appreciated the feeling of camaraderie and reduced isolation (22,35,37). All five studies

reporting on intimate partner violence support groups reported that participants gained a sense of group identity or solidarity (31,40,41,43,44). Lastly, four of the five support groups for mixed injuries reported that they appreciated peer assistance in navigating health and legal systems (23,29,42,45).

Discussion:

We found 25 studies which assessed the benefits of CBPS groups for injured persons, globally. The majority were created for specific types of injury, including spinal cord, brain or head, and brachial plexus injuries as well as burns. There were also groups for intimate partner violence (IPV), torture, and some for mixed injuries. All injury support groups except one were located in high-income countries. Most commonly, participants noted gaining social support, emotional support and catharsis, and educational or didactic materials from CBPS groups. Mental health support was the main reported benefit with few data on improved physical health or retention in care.

Our review identified positive benefits of CBPS for injured people, including group identity, regaining of self-esteem, emotional support and guidance, delivery of practical knowledge about their disease, recovery and treatment process, or ways to access medical, legal, or social support. This mirrors the positive effects of support groups for people living with diabetes (56), mental health problems (57), and heart disease (58), which have been shown to disseminate practical knowledge and health education, social and emotional support, navigation of the medical system, and building trust-based relationships. In addition, the psychological components of both intentional (46) (i.e. interpersonal violence) as well as unintentional (road traffic crashes) injuries can be substantial (47). Further, and highly

applicable to injured patients, peer-led and community-based support groups have implications for rehabilitation, which can reduce limitations in functionality (59) especially in LMICs when access to medical care can be difficult to obtain. CBPS groups have been shown to be useful for retention in care and both physical and mental health improvement in other conditions, and thus offer potential for people who have been injured.

People with injuries potentially face unique challenges in finding peer support compared to people seeking support for specific disease processes, since trauma can result in an array of injuries even given the same mechanism (48). While we found four mixed-injury support groups, twenty-one were injury-specific. Thus, CBPS groups for injured persons are difficult to set up because-persons with injuries may not identify with just one injury type. Furthermore, we did not find support groups for people with certain injury types. For example, there were no studies that reported on CBPS groups for abdominal or thoracic trauma, which can result in significant disability and often is followed by fragmented care in which patients suffer unplanned admissions often at multiple different care sites. This fragmentation ultimately isolates patients and contributes to worse outcomes (49). Additionally, there were no specific CBPS groups for people who have been injured by certain mechanisms, such as road traffic injuries or firearm injury, although these mechanisms are some of the highest contributors to death and disability worldwide (50–52). Victims of these types of trauma might have similar health needs having gone through the same traumatic mechanism, and peer-support could be useful in navigating their care post-injury. These broader groups can also act as powerful advocacy agents; by bringing people with various injuries and mechanisms together, there is a greater opportunity to

advocate for general injury prevention and access to consistent and unfragmented care. (53–55).

We found only one study that was conducted in an LMIC setting. Given the high burden of injury in LMICs where trauma care systems can be especially fragmented resulting in worse access and outcomes for patients (60), there is scope for CBPS groups to help bridge the gaps in these much-needed services. This lack of CBPS in LMICs for people with injuries is congruent with the lack of reporting on the presence of CBPS groups in LMICs aside from those developed for patients who carry diagnoses like HIV/AIDS that have been given prominent status on the global health agenda. Worldwide, peer support has shown efficacy in reducing costs of care, engaging those who are often hard to reach, and providing patient-centered support to empower individuals to manage and direct their care (61). In LMICs, these effects are most often reported in groups that are focused on a defined disease process that are commonly diagnosed in LMIC settings. Twenty-six of the 53 studies in Øgård-Repål et al's review of peer support groups for people living with HIV were undertaken in LMICs (62) and Ayala reported 27 of the 48 studies in a separate scoping review of peer and community led responses to HIV were based in the global south (63). CBPS groups may be especially useful in providing psychosocial, knowledge, and logistical support to those affected by the high burden of injuries in LMICs in addition to ameliorating challenges in healthcare access that delay rehabilitation and medical treatment in these settings.

Gaps in the literature:

The literature is sparse & highly observational in nature, which limits the conclusions that can be drawn on the effectiveness of the groups. However, given the community-based

nature of these types of support groups, it might be difficult to conduct true RCTs. This is reflected in only two RCTs fitting inclusion criteria for our review. Additional observational or survey-based studies might be the only way to discern the effects of the studies. Furthermore, many of the studies that were screened but excluded during this review involved support groups run out of healthcare facilities or under the leadership of healthcare professionals, which shows that while support groups do exist, many still are linked to health care systems.

Limitations:

Our scoping review was limited to articles in the databases we searched and to articles for which we were able to locate full texts. There were 13 (out of 112) articles not found by our search strategy that could have added additional insight. There may be more grey literature available that speaks to the existence and experience of other peer support groups, which was not covered. Additionally, community-based peer support groups in LMICs may be underrepresented because they have not been studied or written about and thus could not be located via our search strategy. This also limits the cultural perspective of our review, as it might not include key aspects of the patient experience in regions other than North America or Europe. Finally, with such a small sample of studies, our conclusions are limited.

Conclusions

Our study shows that community-based support groups play a role in patient recovery and emotional wellbeing after injury. There is a role for information sharing and support that may lead to increased access and retention of care for injured persons. Aside from access and retention in the medical system, support groups can provide practical guidance for

participants in navigating legal systems, as well as in developing connections with other patients and survivors to access to support services. Given the burden of injury that occurs in LMICs, support for development of community-based peer support groups in LMICs may increase access to care and has implications for overall improvement in healthcare delivery.

Table 2: Summary Table of Included Studies

Title	First Author, Year	Country in which the study was conducted	Data collection period	Funding for Group	Study Aim	Study Design	Injury Type	Number of participants	Study Findings
Comparison of a Cognitive-Behavioral Coping Skills Group to a Peer Support Group in a Brain Injury Population	Backhaus, 2016	USA	Not Reported	None	To examine whether group CBT would result in improved perceived self-efficacy (PSE), emotional functioning, and neurobehavioral functioning compared with a structurally similar peer support group program for BI	Randomized control trial	Brain Injury	38	Individuals with BI and their caregivers may benefit from either group CBT or a peer support group, with a trend for those received group CBT to maintain the effects of improved PSE. For our review, we extracted data solely from the control group, which functioned as a support group in its own right.
Helping others heal: burn survivors and peer support	Badger, 2010	US/Canada	Not Reported	None	To evaluate perceptions of peer support and the value they placed on it related to the key burn recovery outcome variables of: social comfort, life satisfaction, QOL (body image, affect, interpersonal relationships, sexuality, productivity, and social integration.	Cross Sectional	Burn	95	Burn survivors held a positive regard for peer support, with it being ranked as the best resource of the Phoenix society.
Adult burn survivors' views of peer support: a qualitative study	Badger, 2010	Canada	2007	None	To learn from burn survivors whether peer support was important to them in their recovery and why, and secondarily, if they had volunteered as a	Qualitative	Burn	30	Burn survivors highly valued peer support, especially by providing hope, combatting isolation, and lessening the feeling of being stigmatized due to physical scars.

					peer supporter, if there were any negative consequences or advice they would have for others.				
**Title**	**First Author, Year**	**Country in which the study was conducted**	**Data collection period**	**Funding for Group**	**Study Aim**	**Study Design**	**Injury Type**	**Number of participants**	**Study Findings**
Processes and outcomes of a communalization of trauma approach: Vets & Friends community-based support groups	Balmer, 2021	USA	2017	None	To inform training and group leader support procedures to ensure the maximal functioning and effectiveness of V&F support groups	Qualitative	Mixed Injury group	23	Outcomes included restoration of trust, connection with group members, building skills to manage trauma, and community acceptance and engagement. It also found that Vets and Friends could possible meeting veteran specific needs.
Making meaning in a burn peer support group: qualitative analysis of attendee interviews	Davis, 2014	USA	2010-2011	None	To better understand the psychosocial recovery needs of burn survivors and the role of peer support groups, by performing a series of guided qualitative interviews.	Qualitative	Burn	6 interviewees, 6 key informants, 30-50 group members	The support group was instrumental to their psychosocial adjustment and growth. It allowed them to make meaning of their injury, learn coping strategies, make meaningful relationships, and have hope.
Constructing robust selves after brain injury: positive identity work among members of a female self-help group	Gelech, 2019	Canada	2012	Saskatchewan Association for the Rehabilitation of the Brain Injured Shoppers Drug Mart Foundation for Women	To understand the work around identity in rehabilitation contexts by analysing how survivors construct the self in a female acute BI survivor self-help group.	Qualitative	Brain Injury	5	Four modes of identity work were seen: constructing competent selves, tempering the threat of loss and impairment, resisting infantilization and delegitimization, and asserting a collective gender identity.

Title	First Author, Year	Country in which the study was conducted	Data collection period	Funding for Group	Study Aim	Study Design	Injury Type	Number of participants	Study Findings
Long-Term Social Reintegration Outcomes for Burn Survivors With and Without Peer Support Attendance: A Life Impact Burn Recovery Evaluation (LIBRE) Study	Grieve, 2020	US/Canada	October 2014 - December 2015	None	To determine the association of peer group attendance with the societal reintegration of a large group of burn survivors from the US and Canada using the Life Impact Burn Recovery Evaluation (LIBRE) profile.	Cross Sectional	Burn	601 responded to survey; 330 participated in peer support	Burn survivors who attended peer support groups had better scores on social iterations, social activities, and work and employment than those who did not. Those who attended also had larger burns and were more likely to be more than 10 years out from injury.
Accessing peers' and health care experts' wisdom: a telephone peer support program for women with SCI living in rural and remote areas	Jalovcic, 2009	Canada	2004	None	To capture the essence of women’s experiences of the Telephone Peer Support Group program and its main characteristics and structure as perceived by participants.	Qualitative	Spinal Cord Injury	7	The participatory approach of this group allowed participants to control the direction of the group, allowing for emotional support and exchange. There were 17 core themes that were grouped into four structures: space, relation to self, relation to others, and causality.
Group therapy for refugees and torture survivors: treatment model innovations	Kira, 2012	USA	2006-2008	None	To develop group therapy models based on thorough and in-depth analyses of the nature of torture and its psychosocial effects through the study of a social organization for	Case Report	Torture/ Refugee	20 women (first group); 8-12 families (2nd group)	Group therapy for torture survivors is an important part of the comprehensive rehabilitation approach for survivors, and that group therapies extend into community healing.

					Bhutanese torture survivors.				
**Title**	**First Author, Year**	**Country in which the study was conducted**	**Data collection period**	**Funding for Group**	**Study Aim**	**Study Design**	**Injury Type**	**Number of participants**	**Study Findings**
Observations from practice: support group membership as a process of social capital formation among female survivors of domestic violence	Larance, 2004	USA	January 2000 - December 2002	Jersey Battered Women's Service, Inc.'s Community Outreach Program	To explore the authors' practice observations of female domestic violence survivors' journey from first agency contact to active participation in a support group process.	Mixed Methods (Participant Observation and Survey Data)	Physical Intimate Partner Violence	412	Support groups provide abused women with the opportunity to build trust and networks, with demonstrations of calling on these relationships outside of the agency's confines for support and enjoyment.
Using an integrated knowledge translation approach to inform a pilot feasibility randomized controlled trial on peer support for individuals with traumatic brain injury: A qualitative descriptive study	Lau, 2021	Canada	2021	None	(1) To understand key informants' perspectives of the barriers and facilitators of participating in peer support research and programs among individuals with TBI; (2) to understand key informants' perspectives on the perceived impacts of peer support programs on individuals with TBI; and, (3) to demonstrate how an iKT approach can inform the development and implementation of a pilot feasibility randomized controlled trial (RCT).	Qualitative	Brain Injury	22	There were five main themes related to the barriers and facilitators to participating in peer support research and programs: knowledge, awareness, and communication; logistics of participating; readiness and motivation to participate; need for clear expectations; and matching. There were three main themes related to the perceived impact of peer support: acceptance, community, social experiences; vicarious experience/learning through others: shared experiences, role-modelling,

									encouragement; and “I feel better.”
**Title**	**First Author, Year**	**Country in which the study was conducted**	**Data collection period**	**Funding for Group**	**Study Aim**	**Study Design**	**Injury Type**	**Number of participants**	**Study Findings**
A Pilot Feasibility Randomized Controlled Trial on the Ontario Brain Injury Association Peer Support Program	Levy, 2021	Canada	2021	None	The overall objective of this study was to determine the feasibility of conducting an RCT and the preliminary effectiveness of the OBIA Peer Support Program	Randomized control trial	Brain Injury	13	The study found no statistically significant results for community integration, mood, or self-efficacy; however, changes in these outcomes were accompanied by moderate-to-large effect sizes. Within health-related quality of life, the mean pain score of the intervention group was significantly lower than that of the control group at the two-month timepoint but not at completion. Interviews revealed proximal improvements in knowledge, skills, and goals, and identified two domains related to trial acceptability: (1) environmental context and resources, and (2) reinforcement.
Unexpected barriers in return to work: lessons learned from injured worker peer support groups	MacEachen, 2007	Canada	2004	None	To study injured worker peer support groups for an empirical picture of return-to-work difficulties, including how peer support performs both positive and negative return-to-work functions at the individual and policy level.	Qualitative	Mixed injury group	37	The study identified four dimensions of peer support: worker experience of being misunderstood by system providers, need for advocates, social support, and help with procedural complexities of the workers’ compensation and health care systems. It concluded

									that peer support groups can be part of the return to work for workers, but there is also a need for more sensitivity to structural and social issues.
**Title**	**First Author, Year**	**Country in which the study was conducted**	**Data collection period**	**Funding for Group**	**Study Aim**	**Study Design**	**Injury Type**	**Number of participants**	**Study Findings**
Support groups for injured workers: process and outcomes	Mignone, 1999	Canada	1992-1994	Workers' Compensation Board of Alberta	This study evaluated the impact of support groups on injured workers with musculoskeletal injuries in relation to pain, somatization, depression, and pain-locus-of-control.	Cross Sectional	Mixed Injury group	62 in intervention; 40 comparison	The study found that between those who did and didn't participate in the support groups, there was no demonstrable effect on the well-being of those who did participate, but it could not rule out any benefits of participating in support groups.
Divorcing abused Latina immigrant women's experiences with domestic violence support groups	Molina, 2009	USA	Not Reported	None	The purpose of this study was to explore and describe the experiences of Latina immigrants who had been members of 1 year-long domestic violence support groups.	Cross Sectional	Physical Intimate Partner Violence	15	Overall the group experience was positive, with members learning about the domestic violence cycle, how to understand their children, their childhood abuse, and legal information on their situation. The members felt like they made friends, felt protected, comfortable, and understood, and gave them courage to fight.

Title	First Author, Year	Country in which the study was conducted	Data collection period	Funding for Group	Study Aim	Study Design	Injury Type	Number of participants	Study Findings
From isolation to connection: understanding a support group for Hispanic women living with gender-based violence in Houston, Texas	Morales, 2009	USA	2005	La Rosa Family Services	To examine how the community based organization's support group encourages immigrant Hispanic women to focus on developing tools and strategies, self-esteem, and empowerment.	Mixed Methods (Archival research, Interviews, & Participant Observation)	Physical Intimate Partner Violence	30	Women enjoyed the benefits of the support group and were taught to manage situations, emotions, and becoming less dependent on the abuser. They also became more aware of their self-worth and value and are making efforts towards self-sufficiency.
A Thematic Analysis of Online Discussion Boards for Brachial Plexus Injury	Morris, 2016	Internet	2015	None	The purpose of this study was to examine posts on Internet discussion groups dedicated to brachial plexus injury and identify common ideas through thematic analysis	Qualitative	Brachial Plexus Injury	336 posts	Themes focused on emotional aspects of brachial plexus injury and information support related to the disease, treatment, recovery after treatment, and process of seeking care.
Helping the patient cope with the sequelae of trauma through the self-help group-approach	Scanlon-Schlipp, 1981	USA	Not Reported	None	To describe the development of Trauma Recovery, a self-help group	Case Report	Mixed injury group	n/a	The self-help group is an effective means for recoverees to engage in therapeutic activities and is cost efficient. The Trauma Recovery program has a psycohoscial emphasis and has established a groundwork for regional chapters.

Title	First Author, Year	Country in which the study was conducted	Data collection period	Funding for Group	Study Aim	Study Design	Injury Type	Number of participants	Study Findings
Helping factors in a peer-developed support group for persons with head injury, Part 2: Survivor interview perspective	Schulz, 1994	USA	1993	Vital Active Life after Trauma Inc	The purpose of the study was to determine the perspective of group members relative to helping factors in their peer-developed support group	Qualitative	Brain Injury	4	Eleven helping factors were generated: socializing, finding out other people's perspectives and attitudes, expressing thoughts and feelings, receiving support, feeling something in common, gaining understanding, empathy and acceptance through listening and sharing, getting perspective, helping others, getting help, feeling hope, and learning more about BI.
Helping factors in a peer-developed support group for persons with head injury, Part 1: Participant observer perspective	Schwartzberg, 1994	USA	Not Reported	Vital Active Life after Trauma Inc	An ethnographic study to identify the helping factors in a peer-developed support group for persons who survived a traumatic head injury.	Qualitative	Brain Injury	13	Positive attributes in a successful peer support group were feeling a part of the group, having a common problem and being able to validate the effects of an injury, and receiving information through a variety of channels.

Title	First Author, Year	Country in which the study was conducted	Data collection period	Funding for Group	Study Aim	Study Design	Injury Type	Number of participants	Study Findings
Promoting group empowerment and self-reliance through participatory research: a case study of people with physical disability	Stewart, 1999	South Africa	Not Reported	None	The purpose of this case study of a SCI self-help group is (1) To share the experiences of both the health professional and group members in moving from an originally professionally-led group to an independent peer-led group (2) To demonstrate how participatory research may serve as a tool for group empowerment and self-reliance; and (3) To illustrate how a critically informed professional group work praxis may enable marginalized groups to take action for social change.	Case Report	Spinal Cord Injury	12	This case study shows how group participation with a participatory research framework can unlock self-reliance and empowerment. It also offers insight to the group process and the role of health professionals to create opportunities for empowerment and self-reliance for people with a disability.
The Effect of a Trauma Risk Management (TRiM) Program on Stigma and Barriers to Help-Seeking in the Police	Watson, 2018	UK	November 2012 - January 2013	None	The primary aim of this study was to investigate differences between Trauma Risk Management (TriM) and non-TriM-using forces on help-seeking and stigma among serving police personnel. In addition, the study explored whether	Cross Sectional	Mixed Injury group	859	The TriM group reported lower levels of PTSD symptoms and showed less stigmatized views towards mental health difficulties. They also percieved fewer barriers to help-seeking.

					TriM had any effect on PTSD symptoms.				
**Title**	**First Author, Year**	**Country in which the study was conducted**	**Data collection period**	**Funding for Group**	**Study Aim**	**Study Design**	**Injury Type**	**Number of participants**	**Study Findings**
Online Conversations About Abuse: Responses to IPV Survivors from Support Communities	Whiting, 2022	Internet	2019	None	In this analysis we sought to discover the types of responses being offered in reply to survivors' self-disclosures and questions regarding their experiences with IPV.	Qualitative	Physical Intimate Partner Violence	451	There were three main categories of responses from the data, including support, sharing experiences, and sharing information. The subcategories included specific types of help, advice, understanding, and were overwhelmingly positive in their tone and content.
The self-help movement in head injury	Williams, 1990	USA	Not Reported	None	A survey to describe self-help groups and their benefits	Cross Sectional	Brain Injury	77	The main focus of meetings was social support. Membership varies by time after injury, sex, and relationship to survivors, and these variables impact how the support group meets the needs of its members. Group leaders should often survey the group to ensure needs are being met. The study suggests that nurses may provide an important role in recruitment, information, and group facilitation.

Title	First Author, Year	Country in which the study was conducted	Data collection period	Funding for Group	Study Aim	Study Design	Injury Type	Number of participants	Study Findings
Support groups for older victims of domestic violence	Wolf, 2001	US/Canada	1997	Domestic violence programs, ageing services	To identify support groups for older victims of abuse, to gather information that would be useful in assisting communities to meet the needs of these elders, and to determine whether the type of sponsorship (aging services or domestic violence programs) made a difference in group operations.	Mixed Methods (cross sectional survey & interviews)	Physical Intimate Partner Violence	30 support groups	Recommendations for future groups include accessibility of the meeting site, using a leader familiar with aging issues or an elder themselves, resources for recruitment, and a steady source of funding. Resistance of elders to participate was noted to be a large barrier.

References:

1. Injuries and violence [Internet]. [cited 2021 Nov 18]. Available from: https://www.who.int/news-room/fact-sheets/detail/injuries-and-violence

2. Alonge O, Agrawal P, Talab A, Rahman QS, Rahman AF, Arifeen SE, et al. Fatal and non-fatal injury outcomes: results from a purposively sampled census of seven rural subdistricts in Bangladesh. The Lancet Global Health. 2017 Aug 1;5(8):e818–27.

3. Whitaker J, Denning M, O'Donohoe N, Poenaru D, Guadagno E, Leather A, et al. Assessing trauma care health systems in low- and middle-income countries, a protocol for a systematic literature review and narrative synthesis. Systematic Reviews. 2019 Jul 2;8(1):157.

4. Min SY, Whitecraft J, Rothbard AB, Salzer MS. Peer Support for Persons with Co-Occurring Disorders and Community Tenure: A Survival Analysis. Psychiatric Rehabilitation Journal. 2007;30(3):207–13.

5. Jordan G, Grazioplene R, Florence A, Hammer P, Funaro MC, Davidson L, et al. Generativity among persons providing or receiving peer or mutual support: A scoping review. Psychiatric Rehabilitation Journal [Internet]. 2021 Oct 7 [cited 2021 Nov 30]; Available from: http://doi.apa.org/getdoi.cfm?doi=10.1037/prj0000498

6. Liang D, Jia R, Zhou X, Lu G, Wu Z, Yu J, et al. The effectiveness of peer support on self-efficacy and self-management in people with type 2 diabetes: A meta-analysis. Patient Educ Couns. 2021 Apr;104(4):760–9.

7. Hossain SN, Jaglal SB, Shepherd J, Perrier L, Tomasone JR, Sweet SN, et al. Web-Based Peer Support Interventions for Adults Living With Chronic Conditions: Scoping Review. JMIR Rehabil Assist Technol. 2021 May 25;8(2):e14321.

8. Shalaby RAH, Agyapong VIO. Peer Support in Mental Health: Literature Review. JMIR Ment Health. 2020 Jun 9;7(6):e15572.

9. Solomon P. Peer Support/Peer Provided Services Underlying Processes, Benefits, and Critical Ingredients. Psychiatric Rehabilitation Journal. 2004;27(4):392–401.

10. Berg RC, Page S, Øgård-Repål A. The effectiveness of peer-support for people living with HIV: A systematic review and meta-analysis. PLoS One. 2021;16(6):e0252623.

11. Kun KE, Couto A, Jobarteh K, Zulliger R, Pedro E, Malimane I, et al. Mozambique's Community Antiretroviral Therapy Support Group Program: The Role of Social Relationships in Facilitating HIV/AIDS Treatment Retention. AIDS Behav. 2019 Sep;23(9):2477–85.

12. McLeish J, Redshaw M. Mothers' accounts of the impact on emotional wellbeing of organised peer support in pregnancy and early parenthood: a qualitative study. BMC Pregnancy Childbirth. 2017 Jan 13;17(1):28.

13. Gruß I, Firemark A, Davidson A. Motherhood, substance use and peer support: Benefits of an integrated group program for pregnant and postpartum women. J Subst Abuse Treat. 2021 Dec;131:108450.

14. O'Mara-Eves A, Brunton G, Oliver S, Kavanagh J, Jamal F, Thomas J. The effectiveness of community engagement in public health interventions for disadvantaged groups: a meta-analysis. BMC Public Health. 2015 Feb 12;15(1):129.

15. Lauckner HM, Hutchinson SL. Peer support for people with chronic conditions in rural areas: a scoping review. Rural Remote Health. 2016 Mar;16(1):3601.

16. Maxwell C, Aggleton P, Warwick I. Involving HIV-positive people in policy and service development: Recent experiences in England. null. 2008 Jan 1;20(1):72–9.

17. Carter A, Greene S, Nicholson V, O'Brien N, Sanchez M, de Pokomandy A, et al. Breaking the Glass Ceiling: Increasing the Meaningful Involvement of Women Living With HIV/AIDS (MIWA) in the Design and Delivery of HIV/AIDS Services. null. 2015 Aug 3;36(8):936–64.

18. Arksey H, O'Malley L. Scoping studies: towards a methodological framework. null. 2005 Feb 1;8(1):19–32.

19. Levac D, Colquhoun H, O'Brien KK. Scoping studies: advancing the methodology. Implementation Science. 2010 Sep 20;5(1):69.

20. Veritas Health Innovation. Covidence systematic review software [Internet]. Melbourne, Australia; Available from: www.covidence.org

21. Backhaus S, Ibarra S, Parrott D, Malec J. Comparison of a Cognitive-Behavioral Coping Skills Group to a Peer Support Group in a Brain Injury Population. Arch Phys Med Rehabil. 2016 Feb;97(2):281–91.

22. Badger K, Royse D. Helping others heal: burn survivors and peer support. Social work in health care. 2010;49(1):1–18.

23. MacEachen E, Kosny A, Ferrier S. Unexpected barriers in return to work: lessons learned from injured worker peer support groups. Work. 2007;29(2):155–64.

24. Jalovcic D, Pentland W. Accessing Peers' and Health Care Experts' Wisdom: A Telephone Peer Support Program for Women with SCI Living in Rural and Remote Areas. Topics in Spinal Cord Injury Rehabilitation. 2009 Aug 16;15(1):59–74.

25. Stewart R, Bhagwanjee A. Promoting group empowerment and self-reliance through participatory research: a case study of people with physical disability. Disabil Rehabil. 1999 Jul;21(7):338–45.

26. Schulz CH. Helping factors in a peer-developed support group for persons with head injury, Part 2: Survivor interview perspective. Am J Occup Ther. 1994 Apr;48(4):305–9.

27. Morris M, Daluiski A, Dy C. A Thematic Analysis of Online Discussion Boards for Brachial Plexus Injury. The Journal of hand surgery [Internet]. 2016 Aug [cited 2023 Mar 9];41(8). Available from: https://pubmed.ncbi.nlm.nih.gov/27311862/

28. Schwartzberg SL. Helping factors in a peer-developed support group for persons with head injury, Part 1: Participant observer perspective. Am J Occup Ther. 1994 Apr;48(4):297–304.

29. Mignone J, Guidotti TL. Support groups for injured workers: process and outcomes. J Occup Environ Med. 1999 Dec;41(12):1059–64.

30. Williams MH. The self-help movement in head injury. Rehabil Nurs. 1990;15(6):311–5.

31. Whiting JB, Davies BN, Eisert BC, Witting AB, Anderson SR. Online Conversations About Abuse: Responses to IPV Survivors from Support Communities. J Fam Violence. 2022 Jul 22;1–11.

32. Levy BB, Luong D, Bayley MT, Sweet SN, Voth J, Kastner M, et al. A Pilot Feasibility Randomized Controlled Trial on the Ontario Brain Injury Association Peer Support Program. J Clin Med. 2021 Jun 29;10(13):2913.

33. Lau SKC, Luong D, Sweet SN, Bayley M, Levy BB, Kastner M, et al. Using an integrated knowledge translation approach to inform a pilot feasibility randomized controlled trial on peer support for individuals with traumatic brain injury: A qualitative descriptive study. PLoS One. 2021;16(8):e0256650.

34. Gelech J, Bayly M, Desjardins M. Constructing robust selves after brain injury: positive identity work among members of a female self-help group. Neuropsychol Rehabil. 2019 Apr;29(3):456–76.

35. Badger K, Royse D. Adult burn survivors' views of peer support: a qualitative study. Soc Work Health Care. 2010;49(4):299–313.

36. Grieve B, Shapiro GD, Wibbenmeyer L, Acton A, Lee A, Marino M, et al. Long-Term Social Reintegration Outcomes for Burn Survivors With and Without Peer Support Attendance: A Life Impact Burn Recovery Evaluation (LIBRE) Study. Arch Phys Med Rehabil. 2020 Jan;101(1S):S92–8.

37. Davis T, Gorgens K, Shriberg J, Godleski M, Meyer L. Making meaning in a burn peer support group: qualitative analysis of attendee interviews. J Burn Care Res. 2014;35(5):416–25.

38. Kira IA, Ahmed A, Wasim F, Mahmoud V, Colrain J, Rai D. Group therapy for refugees and torture survivors: treatment model innovations. Int J Group Psychother. 2012 Jan;62(1):69–88.

39. Scanlon-Schilpp AM, Levesque J. Helping the patient cope with the sequelae of trauma through the self-help group approach. J Trauma. 1981 Feb;21(2):135–9.

40. Molina O, Lawrence SA, Azhar-Miller A, Rivera M. Divorcing Abused Latina Immigrant Women's Experiences With Domestic Violence Support Groups. Journal of Divorce & Remarriage. 2009 Sep 30;50(7):459–71.

41. Morales-Campos DY, Casillas M, McCurdy SA. From isolation to connection: understanding a support group for Hispanic women living with gender-based violence in Houston, Texas. J Immigr Minor Health. 2009 Feb;11(1):57–65.

42. Balmer BR, Sippola J, Beehler S. Processes and outcomes of a communalization of trauma approach: Vets & Friends community-based support groups. J Community Psychol. 2021 Sep;49(7):2764–80.

43. Wolf RS. Support groups for older victims of domestic violence. J Women Aging. 2001;13(4):71–83.

44. Larance L, Porter M. Observations From Practice: Support Group Membership as a Process of Social Capital Formation Among Female Survivors of Domestic Violence | Office of Justice Programs. Journal of Interpersonal Violence. 2004 Jun;19(6):676–90.

45. Watson L, Andrews L. The effect of a Trauma Risk Management (TRiM) program on stigma and barriers to help-seeking in the police. International Journal of Stress Management. 2018;25:348–56.

46. Rivara F, Adhia A, Lyons V, Massey A, Mills B, Morgan E, et al. The Effects Of Violence On Health. Health Affairs. 2019 Oct;38(10):1622–9.

47. Craig A, Tran Y, Guest R, Gopinath B, Jagnoor J, Bryant RA, et al. Psychological impact of injuries sustained in motor vehicle crashes: systematic review and meta-analysis. BMJ Open. 2016 Sep 1;6(9):e011993.

48. Hardy BM, King KL, Enninghorst N, Balogh ZJ. Trends in polytrauma incidence among major trauma admissions. Eur J Trauma Emerg Surg [Internet]. 2022 Dec 19 [cited 2023 Jul 8]; Available from: https://doi.org/10.1007/s00068-022-02200-w

49. Carr MJ, Badiee J, Benham DA, Diaz JA, Calvo RY, Sise CB, et al. Fragmentation of care in the blunt abdominal trauma patient: Capturing our true outcomes and impact on care. J Trauma Acute Care Surg. 2021 Nov 1;91(5):829–33.

50. Nantulya VM, Reich MR. The neglected epidemic: road traffic injuries in developing countries. BMJ. 2002 May 11;324(7346):1139–41.

51. Global Burden of Disease 2016 Injury Collaborators, Naghavi M, Marczak LB, Kutz M, Shackelford KA, Arora M, et al. Global Mortality From Firearms, 1990-2016. JAMA. 2018 Aug 28;320(8):792–814.

52. Rissanen R, Ifver J, Hasselberg M, Berg HY. Quality of life following road traffic injury: the impact of age and gender. Qual Life Res. 2020 Jun 1;29(6):1587–96.

53. Dain K. A new chapter for the NCD Alliance: stronger together. The Lancet. 2019 May 27;389(10084):2089–90.

54. Meara JG, Leather AJM, Hagander L, Alkire BC, Alonso N, Ameh EA, et al. Global Surgery 2030: evidence and solutions for achieving health, welfare, and economic development. The Lancet. 2015;386(9993):569–624.

55. Riddell MA, Edwards N, Thompson SR, Bernabe-Ortiz A, Praveen D, Johnson C, et al. Developing consensus measures for global programs: lessons from the Global Alliance for Chronic Diseases Hypertension research program. Global Health. 2017 Mar 15;13(1):17.

56. Brownson CA, Heisler M. The role of peer support in diabetes care and self-management. Patient. 2009 Mar 1;2(1):5–17.

57. Brooks H, Devereux-Fitzgerald A, Richmond L, Bee P, Lovell K, Caton N, et al. Assessing the effectiveness of social network interventions for adults with a diagnosis of mental health problems: a systematic review and narrative synthesis of impact. Soc Psychiatry Psychiatr Epidemiol. 2022 May;57(5):907–25.

58. Parry M, Watt-Watson J. Peer support intervention trials for individuals with heart disease: a systematic review. Eur J Cardiovasc Nurs. 2010 Mar;9(1):57–67.

59. Wasilewski MB, Rios J, Simpson R, Hitzig SL, Gotlib Conn L, MacKay C, et al. Peer support for traumatic injury survivors: a scoping review. Disabil Rehabil. 2022 Jun 9;1–34.

60. Shanthakumar D, Payne A, Leitch T, Alfa-Wali M. Trauma Care in Low- and Middle-Income Countries. Surg J (N Y). 2021 Oct 22;7(4):e281–5.

61. Fisher EB. Global Evidence for Peer Support: Humanizing Health Care [Internet]. Peers for Progress; National Council of La Raza; 2014 [cited 2023 Mar 21]. Available from: https://www.ipfcc.org/bestpractices/global-evidence-for-peer-support.pdf

62. Øgård-Repål A, Berg RC, Fossum M. Peer Support for People Living With HIV: A Scoping Review. Health Promot Pract. 2023 Jan;24(1):172–90.

63. Ayala G, Sprague L, Merwe LLA van der, Thomas RM, Chang J, Arreola S, et al. Peer- and community-led responses to HIV: A scoping review. PLOS ONE. 2021 Dec 1;16(12):e0260555.

64. Park HY, Kim MJ, Kim JY, Kim S, Choi JY, Kim JH, et al. Could Peer Support Programs Be a Good Resource for Managing the Unmet Needs of Cancer Patients? J Canc Educ. 2019 Oct 1;34(5):950–7.

65. Lyons N, Cooper C, Lloyd-Evans B. A systematic review and meta-analysis of group peer support interventions for people experiencing mental health conditions. BMC Psychiatry. 2021 Jun 23;21(1):315.

66. Arjadi R, Nauta MH, Chowdhary N, Bockting CLH. A systematic review of online interventions for mental health in low and middle income countries: a neglected field. Glob Ment Health (Camb). 2015;2:e12.

SUPPLEMENT 1: SEARCH STRATEGIES BY DATABASE

PubMed / Medline (National Library of Medicine, NCBI)
2,343 records March 1, 2023

("Self-Help Groups"[Mesh:NoExp] OR community engagement[tiab] OR community group*[tiab] OR community support[tiab] OR help group*[tiab] OR patient support[tiab] OR peer support[tiab] OR peer counsel*[tiab] OR support group*[tiab] OR online support[tiab])

AND
("Wounds and Injuries"[Mesh] OR "Accidents"[Mesh] OR "Violence"[Mesh] OR accidental[tiab] OR accidents[tiab] OR burn[tiab] OR burns[tiab] OR domestic abuse[tiab] OR fallers[tiab] OR falls[tiab] OR fractures[tiab] OR gunshot[tiab] OR injuries[tiab] OR injury[tiab] OR injured[tiab] OR partner abuse[tiab] OR physical abuse[tiab] OR spousal abuse[tiab] OR spouse abuse[tiab] OR stabbing*[tiab] OR trauma[tiab] OR traumas[tiab] OR wound[tiab] OR wounds[tiab] OR violence[tiab])

Cumulative Index to Nursing and Allied Health Literature (CINAHL Complete, EBSCOhost)
1077 records, March 1, 2023

Advanced Search

Search Options
remove:
Apply equivalent subjects

select:
Publication Type: Journal Article

MH "Support Groups" OR MH "Peer Counseling"

OR title OR abstract:

"community engagement" OR "community group*" OR "community support" OR "help group*" OR "patient support" OR "peer support" OR "support group*" OR "online support"

AND

MH "Wounds and Injuries+" OR MH "Accidents+" OR MH "Trauma" OR MH "Violence+"

OR title OR abstract

accidental OR accidents OR burn OR burns OR "domestic abuse" OR fallers OR falls OR fractures OR gunshot OR injuries OR injury OR injured OR "partner abuse" OR "physical abuse" OR "spousal abuse" OR "spouse abuse" OR stabbing* OR trauma OR traumas OR wound OR wounds OR violence

Web of Science Core Collection (Clarivate)

1,569 records, March 1, 2023

Editions:
Science Citation Index Expanded
Social Sciences Citation Index

Advanced Search
More Options: Exact Search

#1
(TI=("community engagement" OR "community group*" OR "community support" OR "help group*" OR "patient support" OR "peer support" OR "support group*" OR "online support")) OR AB=("community engagement" OR "community group*" OR "community support" OR "help group*" OR "patient support" OR "peer support" OR "support group*" OR "online support")

AND

(TI=(accidental OR accidents OR burn OR burns OR domestic abuse OR fallers OR falls OR fractures OR gunshot OR injuries OR injury OR injured OR "partner abuse" OR "physical abuse" OR "spousal abuse" OR "spouse abuse" OR stabbing* OR trauma OR traumas OR wound OR wounds OR violence)) OR (AB=(accidental OR accidents OR burn OR burns OR domestic abuse OR fallers OR falls OR fractures OR gunshot OR injuries OR injury OR injured OR "partner abuse" OR "physical abuse" OR "spousal abuse" OR "spouse abuse" OR stabbing* OR trauma OR traumas OR wound OR wounds OR violence))

#1 AND #2

PubMed: 2,343
CINAHL: 1077
Web of Science Core Collection: 1,569

total # of references retrieved across 3 databases March 1, 2023: 4,989

Figure1

Click here to access/download;Figure;Figure1.tif

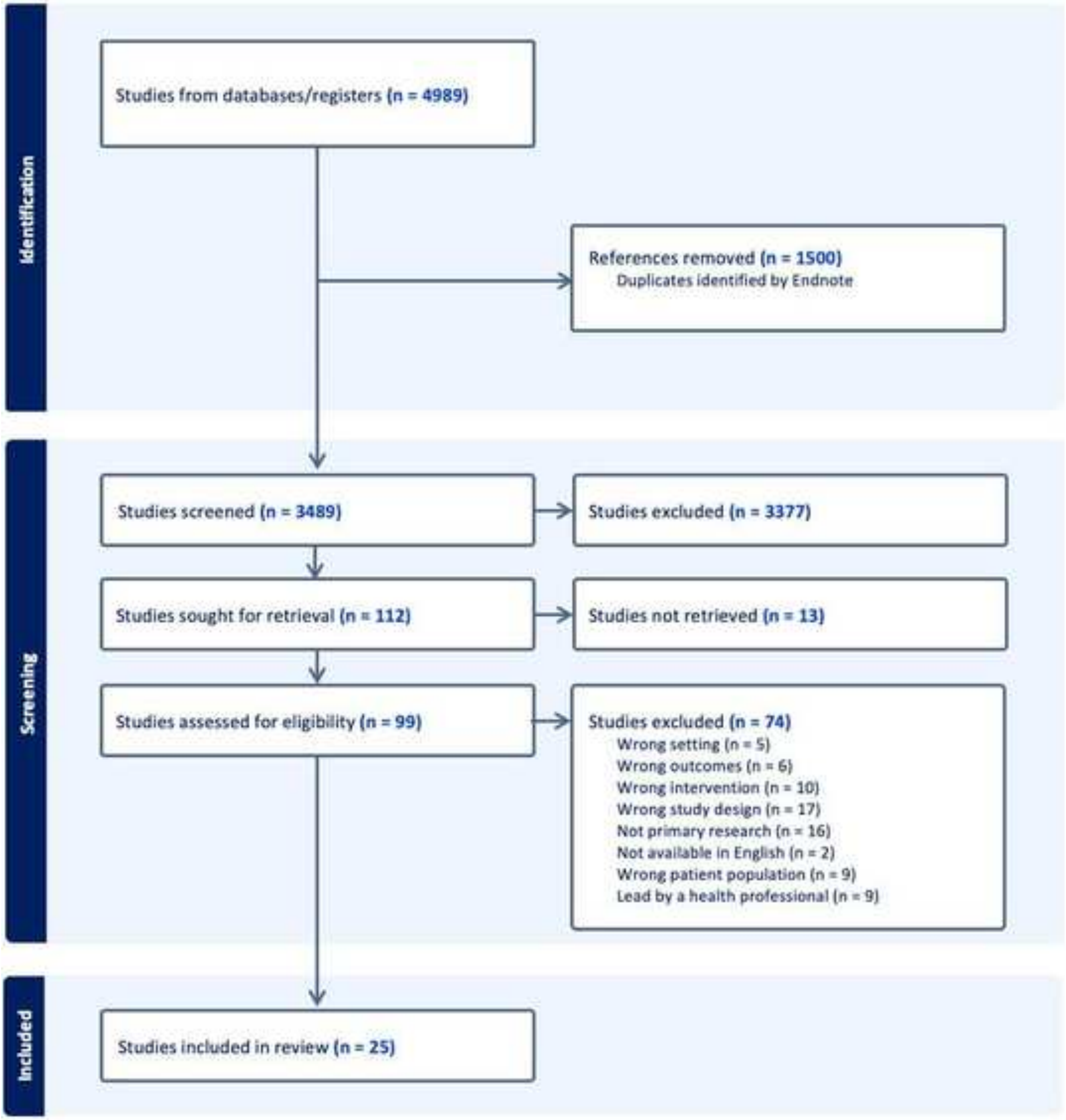

Figure2

Click here to access/download;Figure;Figure2.tif

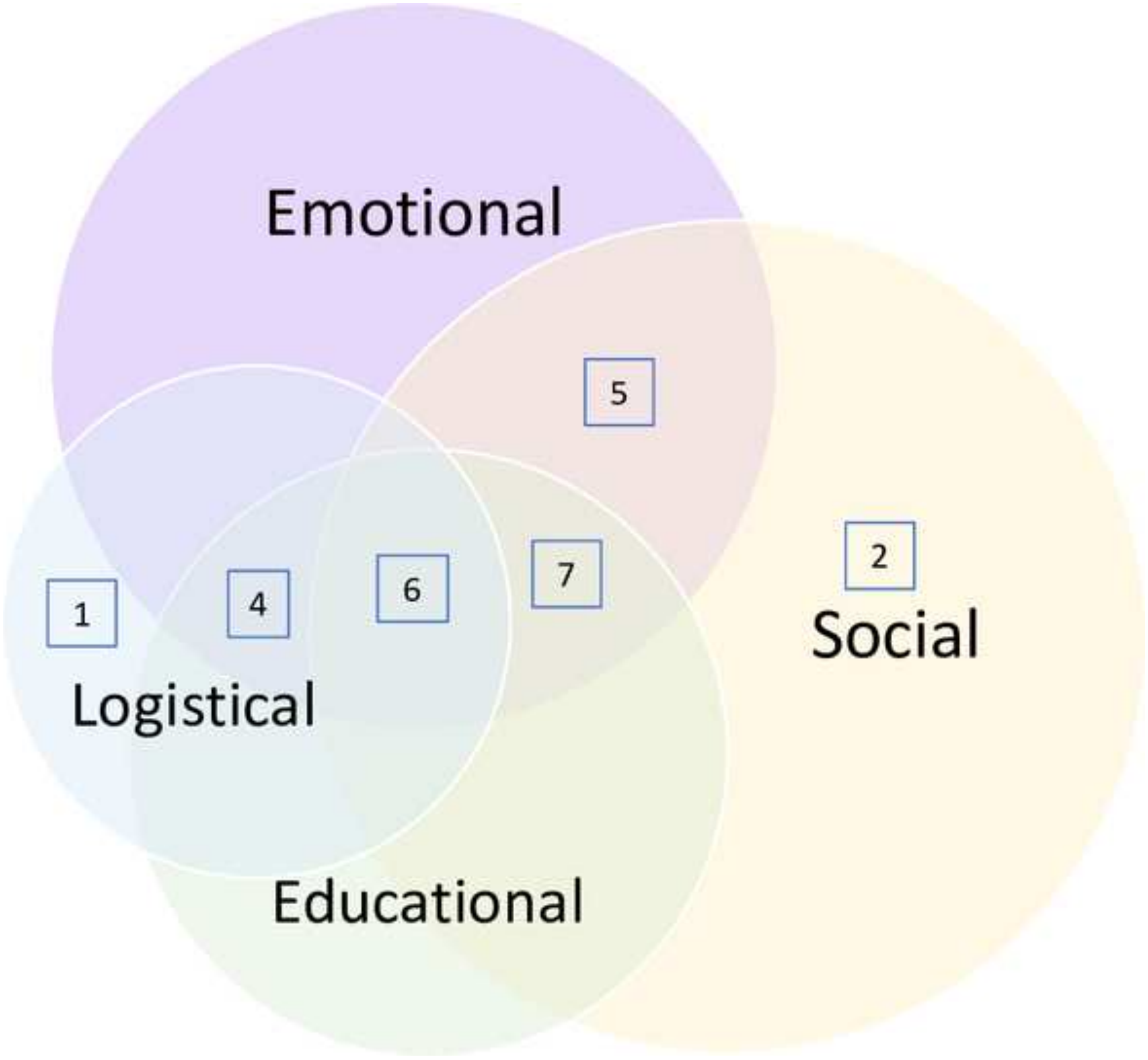

Click here to access/download
Supporting Information
Community Support Injury_PRISMA CHECKLIST.docx

Title: Community Support for Injured Patients: A Scoping Review and Narrative Synthesis

Authors:
Rashi Jhunjhunwala MD MA,[1,2] Anusha Jayaram MD MBA, [1,2] Carol Mita MLIS,[3] Justine Davies MD*[4,5], Kathryn Chu MD MPH FACS FASCRS*[5]
* Joint last authorship

Affiliations:
1. Program in Global Surgery and Social Change, Harvard Medical School, Boston, Massachusetts, USA
2. Department of Surgery, Beth Israel Deaconess Medical Center, Boston, Massachusetts, USA
3. Countway Library, Harvard Medical School, Boston, Massachusetts, USA
4. Institute of Applied Health Research, University of Birmingham, Birmingham, UK
5. Centre for Global Surgery, Department of Global Health, Faculty of Medicine and Health Sciences, Stellenbosch University, Stellenbosch, South Africa

Corresponding Author:
Prof Justine Davies; J.Davies.6@bham.ac.uk

Corresponding Author for Submission:
Rashi Jhunjhunwala; rjhunjhu@bidmc.harvard.edu

Word Count: 3452~~391~~

Abstract:

BACKGROUND: Community-based peer support (CBPS) groups have been effective in facilitating access to and retention in the healthcare system for patients with HIV/AIDS, cancer, diabetes, and other communicable and non-communicable diseases. Given the high incidence of morbidity that results from traumatic injuries, and the barriers to reaching and accessing care for injured patients, community-based support groups may prove to be similarly effective in this population. OBJECTIVES: The objective of this review is to identify the extent and impact of ~~community and peer support groups~~CBPS for injured patients. ELIGIBILITY: We included primary research on studies that evaluated ~~community~~ peer-support groups that were solely based in the community. Hospital-based or healthcare-professional led groups were excluded. EVIDENCE: Sources were identified from a systematic search of Medline / PubMed, CINAHL, and Web of Science Core Collection. CHARTING METHODS: We utilized a narrative synthesis approach to data analysis. RESULTS: 4,989 references were retrieved ~~from database search~~; 25 were included in final data extraction. There was a variety of methodologies represented, ~~,~~ and the groups ~~represented~~ included patients with spinal cord injury (N=2), traumatic brain or head injury (N=7), burns (N=4), intimate partner violence (IPV) (N=5), mixed injuries (N=5), torture (N=1), and brachial plexus injury (N=1). Multiple benefits were reported by support group participants; categorized as social, emotional, logistical, or educational benefits. CONCLUSIONS: community-based peer support groups can provide education, community, and may have implications for retention in care for injured patients.

Introduction:

The World Health Organization (WHO) estimates that 4.4 million people die from injuries such as acts of violence, road traffic accidents, falls, and burns annually, and 90% of these deaths occur in low- and middle-income countries (LMICs) (1). Millions more suffer non-fatal injuries that require extensive medical care and support (2). Injured patients often face many barriers to seeking, reaching, and receiving care (3). These barriers can prevent or delay rehabilitation that allows for return to optimal function, especially after the acute injury has been addressed. Given the potentially chronic conditions that result from injury, retention in care is necessary. However, injured peoples' needs go beyond retention in medical care; they ~~often~~ can also require care for ongoing psychological issues or matters of daily living which are often ~~neglected~~ not addressed within~~by~~ healthcare systems.

For patients with other medical conditions, peer and community support has been shown to facilitate access to healthcare through improved retention of current care, introduction to other providers of care, and provision of information and psychological support (4–7). Peer support has long been used within mental health services and has been defined as social emotional support that is mutually offered or provided by those with similar lived experiences (8). This process of support, companionship, and assistance often counter feelings of loneliness, discrimination, and frustration. The most common form of peer support has been self-help groups, which have been defined as a voluntary small group for mutual aid (9). Studies on peer support amongst HIV patients also demonstrate increased retention in care, improved anti-retroviral therapy adherence and viral suppression, and increased financial and moral support (10,11). Further, studies of pregnant women

demonstrate the positive impact that peer-support can have on motherhood and coping with issues like substance use (12,13). Engaging the community, especially for historically marginalized groups, has also been demonstrated to result in positive health outcomes (14,15). Further, meaningful engagement of and advocacy by members of the HIV/AIDS community has been shown to lead to improved HIV/AIDS services and policy changes that lead to better access to care and service provision (16,17).

Despite the similar needs of injured patients for ongoing medical care and the often life-changing nature of their injuries requiring psychological and physical support, there is little known about whether community-based peer support (CBPS) groups exist, benefit injured persons, or whether they play a role improving and retaining access to acute and chronic injury care. This scoping review aims to identify the ~~extent~~extent~~-~~, distribution, benefits, utility, and impact of community-based ~~and~~ peer support groups for injured patients.

Formatted: Font color: Text 1

Methods:

This scoping review is conducted based on the expanded Arksey and O'Malley framework and uses PRISMA-ScR guidelines (18,19).

Defining the Research Question:

We aimed to answer the question: what research has been done on the ~~use of and benefits~~extent, distribution, benefits, utility, and impact of health-system independent community-based support groups (i.e. those which are not based in healthcare facilities or run by healthcare professionals) for injured persons?

Search Strategy:

Studies reporting on ~~community-based patient support~~CBPS groups for physical injuries were identified by a systematic search of Medline / PubMed (National Library of Medicine, NCBI); CINAHL (CINAHL Complete, EBSCOhost), and Web of Science Core Collection (Clarivate). Controlled vocabulary terms (i.e., MeSH; CINAHL thesaurus subject headings) were included when available and appropriate. The search strategies were designed and carried out by a librarian (CM). No language limits or date restrictions were applied. The exact search terms used for each of the databases are provided in the supplementary document.

These searches were undertaken on March 1st, 2023. We also searched the grey literature via Google searches, reviewed injury society and trauma society web pages, and communicated with global experts in the field of injury research to identify additional studies evaluating the impact of these groups.

Study Inclusion and Exclusion:

Study inclusion and exclusion criteria are shown in **Table 1**. We included primary research studies as well as those which describe advocacy or policy changes to manage injuries, since community support groups have been advocates for improved care for other conditions such as HIV/AIDS (16,17).

We excluded studies that evaluated groups that were hospital or rehabilitation facility-based or led/created by healthcare providers. We also excluded all studies that focused on

Post Traumatic Stress Disorder (PTSD) where it was not clear that the PTSD occurred because of physical injury.

Table 1. Inclusion and exclusion criteria

Inclusion	Exclusion
• All publications through March 1, 2023<br>• Studies of community or patient groups focused on peer-support of people who have been injured and evaluated the impact of community or patient groups for those people<br>• Studies that assess the needs and priorities of community-based patient groups for people who have been injured<br>• All countries<br>• English language	• Studies that evaluated groups that were either led or created by hospital-affiliated staff, healthcare providers, or rehabilitation facilities<br>• Studies of community support groups that focused on injury prevention, self-harm, health issues not related to the injury, or groups that focused only on mental health or Post Traumatic Stress Disorder<br>• Support groups that did not specify that the participants had experienced physical injury

Study Screening & Data Extraction

All studies found were uploaded into Covidence for screening and data extraction. Covidence is a web-based collaboration software platform that streamlines the production of systematic and other literature reviews.(20[illegible]o reviewers (RJ, AJ) screened all titles and abstracts independently for inclusion. In case of disagreement, RJ and AJ resolved conflicts through discussion. This process was repeated for full text review in Covidence. A data extraction form was developed and each of two reviewers independently extracted data from each article, after which consensus was reached through review and discussion. Study data points collected were study design, funding, injury type, the study population, number of participants, what content was shared in the group, and discussion themes in the group. We also collected data on the country in which the support group was based, the country of the study's first and last author, and the years the study was conducted and published.

Data Analysis

We approached the data analysis for this review via a narrative synthesis methodology. This methodology was chosen as it is a widely utilized and accepted mechanism for conducting scoping reviews, as it is a way to synthesize and aggregate the existing body of knowledge on a topic to then elucidate areas of opportunity for further investigation. Since our inclusion criteria did not preclude any specific study methodologies, we expected variety and heterogeneity in our final study sample. We did not assess study quality in this scoping review as we believed all relevant articles per the inclusion criteria should be assessed.

Funding

This study was ~~conducted as~~funded in part ~~of~~ by the Equi-Injury Study, funded by the UK National Institute of Health and Care Research, NIHR Global Health Groups Call 3, application 133135.

Synthesis of Results

The outcomes were grouped into three categories. The first category was study characteristics, which includes study methodology, years during which the studies were conducted, geographic location of support groups, and presence or absence of study funding. We next reported on support group characteristics, including type of injury, number of participants, leadership, and facilitation of the groups, and whether the group itself received funding. Finally, we reported the summary of benefits described by the

support group members – this was categorised by outcomes for all injury types together and then by specific injury type.

Results:

Study Characteristics

4,989 references (PubMed: 2343; CINAHL: 1077; Web of Science: 1569) were retrieved from the database searches on March 1, 2023. Duplicate records were removed using EndNote; import into Covidence resulted in 3489 unique references for screening. Of these, 112 were included for full text review. Full text was not recoverable for 13 studies due to unavailability online through Harvard libraries, or inter-library loan, or other formal retrieval mechanisms. Four of 13 unavailable studies were published prior to 1990 and contact information for the authors was not available. Attempts to contact the other nine authors were unsuccessful. Of the 99 full texts that were reviewed, 25 studies met criteria for data extraction (**Figure 1).**

Figure 1. PRISMA Flow diagram

Study characteristics

Of the 25 studies included, eleven used purely qualitative methodology (23, 24, 26- 28, 31, 33- 35, 37, 42), six were cross-sectional quantitative studies (22, 29, 30, 36, 40, 45), three used mixed-methods (41, 43, 44), three were case reports (25, 38, 39), and there were two randomized controlled trials (21, 32). Studies were published from 1981 to 2022. Eleven studies were conducted in the US (21, 26, 28, 30, 37- 42, 44), seven in Canada (23, 24, 32- 35, 39), and three in both countries (22, 36, 43). There was one study each from the UK (45)

and South Africa (25). There were two studies in which the peer support occurred in the form of online forums and thereby were not limited by geographic region (27, 31). **Table 2** presents an overview of the 25 studies included in data extraction.

Support Group Characteristics

~~Community-based peer support~~CBPS groups have been studied for persons with spinal cord injury (N=2), traumatic brain or head injury (N=7), burns (N=4), intimate partner violence (IPV) (N=5), mixed injuries (N=5), torture (N=1), and brachial plexus injury (N=1)**.** The number of support group members ranged from 2 to 60+, with most of the groups reporting 20-40 active members. Fifteen studies reported on whether group leadership was provided by members themselves or an external facilitator such as professional facilitators, therapists, social workers, or social work or therapy students (21, 24, 25, 28, 30, 32-34, 38-42, 44, 45). Of these fifteen studies, seven (24, 28, 38, 40, 41, 44, 45) reported that the groups were run by professionals rather than participant volunteers, two groups were run by non-professional facilitators (30, 42). Five groups reported that peers volunteered or rotated through leading the groups (21, 25, 32, 34, 39) and one group utilized one-to-one peer mentorship (33). Data about the group facilitators were unavailable or unclear for the remaining studies. Seven peer-support groups reported receiving funding for the groups' activities or meetings – one from the Alberta Worker's Compensation Board (29) and six from philanthropic organizations (26, 28, 34, 41, 43, 44).

Benefits Reported by Support Group Participants

There were a variety of positive benefits reported by support group members. These were grouped into educational benefits, social benefits, emotional benefits, and logistical benefits

(Figure 2). Educational benefits include explanations and descriptions of various disease processes and what participants could expect for their disease course. Social benefits were defined as those benefits that ameliorated the participants' social network, solidarity with others with similar lived experiences, and community building. Emotional benefits are those benefits that participants indicated were impactful to their sense of self or emotional safety and wellbeing. Lastly, logistical benefits are those that directly pertain to making connections with aid services, legal assistance, guidance on workers' compensation claims, and other administrative issues.

Figure 2. Categories of peer support reported in all studies; number of studies per grouping indicated within boxes.

Thirteen of 25 studies concluded that shared educational or didactic material in the group was a benefit of participation (21–33). Eight studies reported that they valued and appreciated the sharing of medical knowledge, either via dissemination from the group leadership or between participants within the groups (24,26–30,32,34).

From a social perspective, nineteen studies reported that support group members indicated they were able to obtain guidance and coping strategies as a result of group participation (21,23–26,28–33,35–42), and fourteen studies reported that the groups created a sense of solidarity and group identity that members found lacking in other areas of their lives (24,26,30,33–35,37–39,41–44,25). Social interaction was mentioned as a positive outcome in eleven studies (22–24,26,32,33,36,38,40,42,44).

Emotional outcomes constituted a substantial portion of the reported benefits. Fourteen of the studies reported that participants found emotional support and/or catharsis as a

function of group membership (21,24,26–29,31–33,38–42), and fourteen studies reported that group members felt a sense of not feeling alone (22–24,28,29,31,33–35,37,39,40,42,44) after they joined the group. Group participants in eleven studies reported a benefit to their self-esteem, empowerment, and overall quality of life (22,24,25,32,34,35,37,38,40–42). Six studies noted that support groups gave participants a sense of hope for the future (26,32,35,37,39,42). Finally, seven studies reported that participants felt a sense of altruism and being able to give back to other injury survivors as a result of group participation (25,26,28,30,35,37,39). On a ~~practical~~ logistical level, eleven studies reported that group members found that they were able to gain assistance in navigating the health, legal, and/or social systems from their peers (23,26–30,32,40–42,45).

We also evaluated the types of benefits reported by groups for people with specific types of injuries. We focused on studies reporting on brain injury support groups, burn injury support groups, intimate partner violence (IPV) support groups, and mixed injury support groups, as these injury types were the subject of more than two studies included in this review. Table 3 shows the distribution of each type of support reported by each injury group.

Table 3. Peer support type reported by injury group

**Injury type**	Total # of Groups	Emotional support	Educational support	Social support	Logistical support
Brain injury	7	7	7	7	4
Burn injury	4	3	1	4	0
IPV	5	4	1	5	2
Mixed injury	5	4	2	4	4

Considering the types of benefits reported by people with specific types of injuries, out of the seven studies reporting on brain injury support groups, six studies mentioned that

participants ~~found support in the form of~~ guidance and coping strategies from the groups (21,26,28,30,32,33). Of the four studies reporting on ~~patients~~ persons with burn injuries, three reported that participants appreciated t~~the feeling of not being~~he feeling of camaraderie ~~alone that they received from the group~~ and reduced isolation (22,35,37). All five studies reporting on intimate partner violence support groups reported that participants gained a sense of group identity or solidarity (31,40,41,43,44). Lastly, four of the five support groups for mixed injuries reported that they appreciated peer assistance in navigating health and legal systems (23,29,42,45).

Discussion:

We found 25 studies which assessed the benefits of ~~community-based peer support~~CBPS groups for injured persons, globally. The majority were created for specific types of injury, including spinal cord, brain or head, and brachial plexus injuries as well as burns. There were also groups for intimate partner violence (IPV), torture, and some for mixed injuries. All injury support groups except one were located in high-income countries. Most commonly, participants noted gaining social support, emotional support and catharsis, and educational or didactic materials from CBPS groups. Mental health support was the main reported benefit with few data on improved physical health or retention in care.

Our review identified positive benefits of ~~community support groups~~CBPS for injured people, including group identity, regaining of self-esteem, emotional support and guidance, delivery of practical knowledge about their disease, recovery and treatment process, or ways to access medical, legal, or social support. This mirrors the positive effects of support

groups for people living with diabetes (56), mental health problems (57), and heart disease (58), which have been shown to disseminate practical knowledge and health education, social and emotional support, navigation of the medical system, and building trust-based relationships. In addition, the psychological components of both intentional (46) (i.e. interpersonal violence) as well as unintentional (road traffic crashes) injuries can be substantial (47). Further, and highly applicable to injured patients, peer-led and community-based support groups have implications for rehabilitation, which can reduce limitations in functionality (59) especially in LMICs when access to medical care can be difficult to obtain. ~~Community-based peer support~~CBPS groups have been shown to be useful for retention in care and both physical and mental health improvement in other conditions, and thus offer potential for people who have been injured.

People with injuries potentially face unique challenges in finding peer support compared to people seeking support for specific disease processes, since trauma can result in an array of injuries even given the same mechanism (48). While we found four mixed-injury support groups, twenty-one were injury-specific. Thus, CBPS groups for injured persons are difficult to set up because-persons with injuries may not identify with just one injury type. Furthermore, we did not find support groups for people with certain injury types. For example, there were no studies that reported on CBPS groups for abdominal or thoracic trauma, which can result in significant disability and often is followed by fragmented care in which patients suffer unplanned admissions often at multiple different care sites. This fragmentation ultimately isolates patients and contributes to worse outcomes (49). Additionally, there were no specific CBPS groups for people who have been injured by certain mechanisms, such as road traffic injuries or firearm injury, although these

mechanisms are some of the highest contributors to death and disability worldwide (50–52). Victims of these types of trauma might have similar health needs having gone through the same traumatic mechanism, and peer-support could be useful in navigating their care post-injury. These broader groups can also act as powerful advocacy agents; by bringing people with various injuries and mechanisms together, there is a greater opportunity to advocate for general injury prevention and access to consistent and unfragmented care. (53–55).

Formatted: Font color: Text 1

~~Injury, especially in males under 30 years of age, is responsible for a significant portion of illness around the world; the need to be retained in care for chronic disabilities that may result from injuries is essential for maximising health and well-being. In addition, the psychological components of both intentional (46) (i.e. interpersonal violence) as well as unintentional (road traffic crashes) injuries can be substantial (47). Community-based peer support groups have been shown to be useful for retention in care and both physical and mental health improvement in other conditions, and thus offer potential for people who have been injured. This scoping review shows there are few community-based peer support groups offered for patients with injuries, and although these studies report on mental health benefits of peer support, there is little data on whether they improve physical health or retention in care.~~

Formatted: Font color: Black

~~People with traumatic injuries potentially face a unique challenge in finding peer support after their injury as injured patients often experience multiple types of injuries in the same event resulting in a broad range of outcomes (48). This means that injuries do not easily fit into specific disease-based support groups, which is evident even in the array and~~

distribution of community-based peer support groups identified in this review. While there were four support groups for those with mixed injuries, twenty-one of the groups for which injury-related support groups exist focus on a specific type of injury with which prospective participants could identify. This distribution of injury groups which we found in our review does not represent the full spectrum of injury types. For example, there were no studies which reported on groups that focused on people with abdominal or thoracic trauma, which can result in significant disability and often is followed by fragmentation of care (49). Additionally, there were no specific peer support groups identified for those with mechanism-based injuries, such as road traffic injuries or firearm injury, although these mechanisms are some of the highest contributors to death and disability worldwide (50–52). This fragmentation of support groups that we found means that many people with injuries will miss out on opportunities for support. It also means that there is less of an opportunity for groups to advocate for general injury prevention or access to care, if they are limited to individual types of injuries. Whereas, it is known that there is power in bringing fragmented groups together (53–55).

The studies in this review identified positive benefits of community support groups for injured people, including creation of a group identity and regaining of self-esteem, emotional support and guidance, and delivery of practical knowledge about their disease, recovery and treatment process, or ways to access medical, legal, or social support. Several studies reported multiple types of benefit. This mirrors the positive effects of support groups for people living with diabetes (56), mental health problems (57), and heart disease (58), where support groups have been shown to help with sharing of practical knowledge and health education, social and emotional support, navigation of the medical system, and

building trust-based relationships. Further, and highly applicable to injured patients, peer-led and community-based support groups have implications for rehabilitation, which can reduce limitations in functionality (59) especially in LMICs when access to medical care can be difficult to obtain.

We found only one study that was conducted in an LMIC setting. Given the high burden of injury in LMICs where trauma care systems can be especially fragmented resulting in worse access and outcomes for patients (60), there is scope for community-based peer supportCBPS groups to help bridge the gaps in these much-needed services. This lack of CBPSCBSP in LMICs for people with injuries injury is congruent with There is athe lack of reporting on the presence of these types ofCBPS groups in LMICs aside from those developed for patients who carry diagnoses like HIV/AIDS that have been given prominent status on the global health agenda. Worldwide, pPeer support has shown efficacy in reducing costs of care, engaging those who are often hard to reach, and providing patient-centered support to empower individuals to manage and direct their care (61). In LMICs, these effects are most often reported in groups that are focused on a more narrow ora defined disease process that are commonly diagnosed in LMIC settings. Twenty-six of the 53 studies in Øgård-Repål et al’s review of peer support groups for people living with HIV were undertaken in LMICs (62) and Ayala reported 27 of the 48 studies in a separate scoping review of peer and community led responses to HIV were based in the global south (63). Community-based peer supportCBPS groups may be especially useful in providing psychosocial, knowledge, and logistical support to those affected by the high burden of injuries in LMICs in addition to ameliorating challenges in healthcare access that delay rehabilitation and medical treatment in these settings.

Formatted: Font: (Default) +Body (Calibri)

Formatted: Font: (Default) +Body (Calibri)

Formatted: Font: (Default) Calibri

Formatted: Font: (Default) Calibri

Formatted: Font: (Default) Calibri

Gaps in the literature:

The literature is sparse & highly observational in nature, which limits the conclusions that can be drawn on the effectiveness of the groups. However, ~~as~~ given the community-based nature of these types of support groups ~~at the center of this topic~~, it ~~would~~ ~~is~~might be difficult to conduct ~~any~~ true RCTs. This is reflected in ~~as evidenced by~~ only two RCTs fitting inclusion criteria for our review. Additional observational or survey-based studies might be the only way to discern the effects of the studies. Furthermore, many of the studies that were screened but excluded during this review involved support groups run out of healthcare facilities or under the leadership of healthcare professionals, which shows that while support groups do exist, many still are linked to health care systems.

Limitations:

Our scoping review was limited to articles in the databases we searched and to articles for which we were able to locate full texts. There were 13 (out of 112) articles not found by our search strategy that could have added additional insight. There may be more grey literature available that speaks to the existence and experience of other peer support groups, which was not covered. ~~in our review.~~ Additionally, community-based peer support groups in LMICs may be underrepresented because they have not been studied or written about and thus could not be located via our search strategy. This also limits the cultural perspective of our review, as it might not include key aspects of the patient experience in regions other than North America or Europe. Finally, with such a small sample of studies, our conclusions are limited.

Conclusions

Our study shows that community-based support groups play a role in patient recovery and emotional wellbeing after injury. There is a role for information sharing and support that may lead to increased access and retention of care for injured persons. Aside from access and retention in the medical system, support groups can provide practical guidance for participants in navigating legal systems, as well as in developing connections with other patients and survivors to access to support services. Given the burden of injury that occurs in LMICs, support for development of community-based peer support groups in LMICs may increase access to care and has implications for overall improvement in healthcare delivery.

Table 2: Summary Table of Included Studies

Title	First Author, Year	Country in which the study was conducted	Data collection period	Funding for Group	Study Aim	Study Design	Injury Type	Number of participants	Study Findings
Comparison of a Cognitive-Behavioral Coping Skills Group to a Peer Support Group in a Brain Injury Population	Backhaus, 2016	USA	Not Reported	None	To examine whether group CBT would result in improved perceived self-efficacy (PSE), emotional functioning, and neurobehavioral functioning compared with a structurally similar peer support group program for BI	Randomized control trial	Brain Injury	38	Individuals with BI and their caregivers may benefit from either group CBT or a peer support group, with a trend for those received group CBT to maintain the effects of improved PSE. For our review, we extracted data solely from the control group, which functioned as a support group in its own right.
Helping others heal: burn survivors and peer support	Badger, 2010	US/Canada	Not Reported	None	To evaluate perceptions of peer support and the value they placed on it related to the key burn recovery outcome variables of: social comfort, life satisfaction, QOL (body image, affect, interpersonal relationships, sexuality, productivity, and social integration.	Cross Sectional	Burn	95	Burn survivors held a positive regard for peer support, with it being ranked as the best resource of the Phoenix society.
Adult burn survivors' views of peer support: a qualitative study	Badger, 2010	Canada	2007	None	To learn from burn survivors whether peer support was important to them in their recovery and why, and secondarily, if they had volunteered as a	Qualitative	Burn	30	Burn survivors highly valued peer support, especially by providing hope, combatting isolation, and lessening the feeling of being stigmatized due to physical scars.

					peer supporter, if there were any negative consequences or advice they would have for others.				
**Title**	**First Author, Year**	**Country in which the study was conducted**	**Data collection period**	**Funding for Group**	**Study Aim**	**Study Design**	**Injury Type**	**Number of participants**	**Study Findings**
Processes and outcomes of a communalization of trauma approach: Vets & Friends community-based support groups	Balmer, 2021	USA	2017	None	To inform training and group leader support procedures to ensure the maximal functioning and effectiveness of V&F support groups	Qualitative	Mixed Injury group	23	Outcomes included restoration of trust, connection with group members, building skills to manage trauma, and community acceptance and engagement. It also found that Vets and Friends could possible meeting veteran specific needs.
Making meaning in a burn peer support group: qualitative analysis of attendee interviews	Davis, 2014	USA	2010-2011	None	To better understand the psychosocial recovery needs of burn survivors and the role of peer support groups, by performing a series of guided qualitative interviews.	Qualitative	Burn	6 interviewees, 6 key informants, 30-50 group members	The support group was instrumental to their psychosocial adjustment and growth. It allowed them to make meaning of their injury, learn coping strategies, make meaningful relationships, and have hope.
Constructing robust selves after brain injury: positive identity work among members of a female self-help group	Gelech, 2019	Canada	2012	Saskatchewan Association for the Rehabilitation of the Brain Injured Shoppers Drug Mart Foundation for Women	To understand the work around identity in rehabilitation contexts by analysing how survivors construct the self in a female acute BI survivor self-help group.	Qualitative	Brain Injury	5	Four modes of identity work were seen: constructing competent selves, tempering the threat of loss and impairment, resisting infantilization and delegitimization, and asserting a collective gender identity.

Title	First Author, Year	Country in which the study was conducted	Data collection period	Funding for Group	Study Aim	Study Design	Injury Type	Number of participants	Study Findings
Long-Term Social Reintegration Outcomes for Burn Survivors With and Without Peer Support Attendance: A Life Impact Burn Recovery Evaluation (LIBRE) Study	Grieve, 2020	US/Canada	October 2014 - December 2015	None	To determine the association of peer group attendance with the societal reintegration of a large group of burn survivors from the US and Canada using the Life Impact Burn Recovery Evaluation (LIBRE) profile.	Cross Sectional	Burn	601 responded to survey; 330 participated in peer support	Burn survivors who attended peer support groups had better scores on social iterations, social activities, and work and employment than those who did not. Those who attended also had larger burns and were more likely to be more than 10 years out from injury.
Accessing peers' and health care experts' wisdom: a telephone peer support program for women with SCI living in rural and remote areas	Jalovcic, 2009	Canada	2004	None	To capture the essence of women's experiences of the Telephone Peer Support Group program and its main characteristics and structure as perceived by participants.	Qualitative	Spinal Cord Injury	7	The participatory approach of this group allowed participants to control the direction of the group, allowing for emotional support and exchange. There were 17 core themes that were grouped into four structures: space, relation to self, relation to others, and causality.
Group therapy for refugees and torture survivors: treatment model innovations	Kira, 2012	USA	2006-2008	None	To develop group therapy models based on thorough and in-depth analyses of the nature of torture and its psychosocial effects through the study of a social organization for	Case Report	Torture/ Refugee	20 women (first group); 8-12 families (2nd group)	Group therapy for torture survivors is an important part of the comprehensive rehabilitation approach for survivors, and that group therapies extend into community healing.

					Bhutanese torture survivors.				
**Title**	**First Author, Year**	**Country in which the study was conducted**	**Data collection period**	**Funding for Group**	**Study Aim**	**Study Design**	**Injury Type**	**Number of participants**	**Study Findings**
Observations from practice: support group membership as a process of social capital formation among female survivors of domestic violence	Larance, 2004	USA	January 2000 - December 2002	Jersey Battered Women's Service, Inc.'s Community Outreach Program	To explore the authors' practice observations of female domestic violence survivors' journey from first agency contact to active participation in a support group process.	Mixed Methods (Participant Observation and Survey Data)	Physical Intimate Partner Violence	412	Support groups provide abused women with the opportunity to build trust and networks, with demonstrations of calling on these relationships outside of the agency's confines for support and enjoyment.
Using an integrated knowledge translation approach to inform a pilot feasibility randomized controlled trial on peer support for individuals with traumatic brain injury: A qualitative descriptive study	Lau, 2021	Canada	2021	None	(1) To understand key informants' perspectives of the barriers and facilitators of participating in peer support research and programs among individuals with TBI; (2) to understand key informants' perspectives on the perceived impacts of peer support programs on individuals with TBI; and, (3) to demonstrate how an iKT approach can inform the development and implementation of a pilot feasibility randomized controlled trial (RCT).	Qualitative	Brain Injury	22	There were five main themes related to the barriers and facilitators to participating in peer support research and programs: knowledge, awareness, and communication; logistics of participating; readiness and motivation to participate; need for clear expectations; and matching. There were three main themes related to the perceived impact of peer support: acceptance, community, social experiences; vicarious experience/learning through others: shared experiences, role-modelling,

									encouragement; and "I feel better."
**Title**	**First Author, Year**	**Country in which the study was conducted**	**Data collection period**	**Funding for Group**	**Study Aim**	**Study Design**	**Injury Type**	**Number of participants**	**Study Findings**
A Pilot Feasibility Randomized Controlled Trial on the Ontario Brain Injury Association Peer Support Program	Levy, 2021	Canada	2021	None	The overall objective of this study was to determine the feasibility of conducting an RCT and the preliminary effectiveness of the OBIA Peer Support Program	Randomized control trial	Brain Injury	13	The study found no statistically significant results for community integration, mood, or self-efficacy; however, changes in these outcomes were accompanied by moderate-to-large effect sizes. Within health-related quality of life, the mean pain score of the intervention group was significantly lower than that of the control group at the two-month timepoint but not at completion. Interviews revealed proximal improvements in knowledge, skills, and goals, and identified two domains related to trial acceptability: (1) environmental context and resources, and (2) reinforcement.
Unexpected barriers in return to work: lessons learned from injured worker peer support groups	MacEachen, 2007	Canada	2004	None	To study injured worker peer support groups for an empirical picture of return-to-work difficulties, including how peer support performs both positive and negative return-to-work functions at the individual and policy level.	Qualitative	Mixed injury group	37	The study identified four dimensions of peer support: worker experience of being misunderstood by system providers, need for advocates, social support, and help with procedural complexities of the workers' compensation and health care systems. It concluded

									that peer support groups can be part of the return to work for workers, but there is also a need for more sensitivity to structural and social issues.
**Title**	**First Author, Year**	**Country in which the study was conducted**	**Data collection period**	**Funding for Group**	**Study Aim**	**Study Design**	**Injury Type**	**Number of participants**	**Study Findings**
Support groups for injured workers: process and outcomes	Mignone, 1999	Canada	1992-1994	Workers' Compensation Board of Alberta	This study evaluated the impact of support groups on injured workers with musculoskeletal injuries in relation to pain, somatization, depression, and pain-locus-of-control.	Cross Sectional	Mixed Injury group	62 in intervention; 40 comparison	The study found that between those who did and didn't participate in the support groups, there was no demonstrable effect on the well-being of those who did participate, but it could not rule out any benefits of participating in support groups.
Divorcing abused Latina immigrant women's experiences with domestic violence support groups	Molina, 2009	USA	Not Reported	None	The purpose of this study was to explore and describe the experiences of Latina immigrants who had been members of 1 year-long domestic violence support groups.	Cross Sectional	Physical Intimate Partner Violence	15	Overall the group experience was positive, with members learning about the domestic violence cycle, how to understand their children, their childhood abuse, and legal information on their situation. The members felt like they made friends, felt protected, comfortable, and understood, and gave them courage to fight.

Title	First Author, Year	Country in which the study was conducted	Data collection period	Funding for Group	Study Aim	Study Design	Injury Type	Number of participants	Study Findings
From isolation to connection: understanding a support group for Hispanic women living with gender-based violence in Houston, Texas	Morales, 2009	USA	2005	La Rosa Family Services	To examine how the community based organization's support group encourages immigrant Hispanic women to focus on developing tools and strategies, self-esteem, and empowerment.	Mixed Methods (Archival research, Interviews, & Participant Observation)	Physical Intimate Partner Violence	30	Women enjoyed the benefits of the support group and were taught to manage situations, emotions, and becoming less dependent on the abuser. They also became more aware of their self-worth and value and are making efforts towards self-sufficiency.
A Thematic Analysis of Online Discussion Boards for Brachial Plexus Injury	Morris, 2016	Internet	2015	None	The purpose of this study was to examine posts on Internet discussion groups dedicated to brachial plexus injury and identify common ideas through thematic analysis	Qualitative	Brachial Plexus Injury	336 posts	Themes focused on emotional aspects of brachial plexus injury and information support related to the disease, treatment, recovery after treatment, and process of seeking care.
Helping the patient cope with the sequelae of trauma through the self-help group-approach	Scanlon-Schlipp, 1981	USA	Not Reported	None	To describe the development of Trauma Recovery, a self-help group	Case Report	Mixed injury group	n/a	The self-help group is an effective means for recoverees to engage in therapeutic activities and is cost efficient. The Trauma Recovery program has a psycohoscial emphasis and has established a groundwork for regional chapters.

Title	First Author, Year	Country in which the study was conducted	Data collection period	Funding for Group	Study Aim	Study Design	Injury Type	Number of participants	Study Findings
Helping factors in a peer-developed support group for persons with head injury, Part 2: Survivor interview perspective	Schulz, 1994	USA	1993	Vital Active Life after Trauma Inc	The purpose of the study was to determine the perspective of group members relative to helping factors in their peer-developed support group	Qualitative	Brain Injury	4	Eleven helping factors were generated: socializing, finding out other people's perspectives and attitudes, expressing thoughts and feelings, receiving support, feeling something in common, gaining understanding, empathy and acceptance through listening and sharing, getting perspective, helping others, getting help, feeling hope, and learning more about BI.
Helping factors in a peer-developed support group for persons with head injury, Part 1: Participant observer perspective	Schwartzberg, 1994	USA	Not Reported	Vital Active Life after Trauma Inc	An ethnographic study to identify the helping factors in a peer-developed support group for persons who survived a traumatic head injury.	Qualitative	Brain Injury	13	Positive attributes in a successful peer support group were feeling a part of the group, having a common problem and being able to validate the effects of an injury, and receiving information through a variety of channels.

Title	First Author, Year	Country in which the study was conducted	Data collection period	Funding for Group	Study Aim	Study Design	Injury Type	Number of participants	Study Findings
Promoting group empowerment and self-reliance through participatory research: a case study of people with physical disability	Stewart, 1999	South Africa	Not Reported	None	The purpose of this case study of a SCI self-help group is (1) To share the experiences of both the health professional and group members in moving from an originally professionally-led group to an independent peer-led group (2) To demonstrate how participatory research may serve as a tool for group empowerment and self-reliance; and (3) To illustrate how a critically informed professional group work praxis may enable marginalized groups to take action for social change.	Case Report	Spinal Cord Injury	12	This case study shows how group participation with a participatory research framework can unlock self-reliance and empowerment. It also offers insight to the group process and the role of health professionals to create opportunities for empowerment and self-reliance for people with a disability.
The Effect of a Trauma Risk Management (TRiM) Program on Stigma and Barriers to Help-Seeking in the Police	Watson, 2018	UK	November 2012 - January 2013	None	The primary aim of this study was to investigate differences between Trauma Risk Management (TriM) and non-TriM-using forces on help-seeking and stigma among serving police personnel. In addition, the study explored whether	Cross Sectional	Mixed Injury group	859	The TriM group reported lower levels of PTSD symptoms and showed less stigmatized views towards mental health difficulties. They also percieved fewer barriers to help-seeking.

					TriM had any effect on PTSD symptoms.				
**Title**	**First Author, Year**	**Country in which the study was conducted**	**Data collection period**	**Funding for Group**	**Study Aim**	**Study Design**	**Injury Type**	**Number of participants**	**Study Findings**
Online Conversations About Abuse: Responses to IPV Survivors from Support Communities	Whiting, 2022	Internet	2019	None	In this analysis we sought to discover the types of responses being offered in reply to survivors' self-disclosures and questions regarding their experiences with IPV.	Qualitative	Physical Intimate Partner Violence	451	There were three main categories of responses from the data, including support, sharing experiences, and sharing information. The subcategories included specific types of help, advice, understanding, and were overwhelmingly positive in their tone and content.
The self-help movement in head injury	Williams, 1990	USA	Not Reported	None	A survey to describe self-help groups and their benefits	Cross Sectional	Brain Injury	77	The main focus of meetings was social support. Membership varies by time after injury, sex, and relationship to survivors, and these variables impact how the support group meets the needs of its members. Group leaders should often survey the group to ensure needs are being met. The study suggests that nurses may provide an important role in recruitment, information, and group facilitation.

Title	First Author, Year	Country in which the study was conducted	Data collection period	Funding for Group	Study Aim	Study Design	Injury Type	Number of participants	Study Findings
Support groups for older victims of domestic violence	Wolf, 2001	US/Canada	1997	Domestic violence programs, ageing services	To identify support groups for older victims of abuse, to gather information that would be useful in assisting communities to meet the needs of these elders, and to determine whether the type of sponsorship (aging services or domestic violence programs) made a difference in group operations.	Mixed Methods (cross sectional survey & interviews)	Physical Intimate Partner Violence	30 support groups	Recommendations for future groups include accessibility of the meeting site, using a leader familiar with aging issues or an elder themselves, resources for recruitment, and a steady source of funding. Resistance of elders to participate was noted to be a large barrier.

References:

1. Injuries and violence [Internet]. [cited 2021 Nov 18]. Available from: https://www.who.int/news-room/fact-sheets/detail/injuries-and-violence

2. Alonge O, Agrawal P, Talab A, Rahman QS, Rahman AF, Arifeen SE, et al. Fatal and non-fatal injury outcomes: results from a purposively sampled census of seven rural subdistricts in Bangladesh. The Lancet Global Health. 2017 Aug 1;5(8):e818–27.

3. Whitaker J, Denning M, O'Donohoe N, Poenaru D, Guadagno E, Leather A, et al. Assessing trauma care health systems in low- and middle-income countries, a protocol for a systematic literature review and narrative synthesis. Systematic Reviews. 2019 Jul 2;8(1):157.

4. Min SY, Whitecraft J, Rothbard AB, Salzer MS. Peer Support for Persons with Co-Occurring Disorders and Community Tenure: A Survival Analysis. Psychiatric Rehabilitation Journal. 2007;30(3):207–13.

5. Jordan G, Grazioplene R, Florence A, Hammer P, Funaro MC, Davidson L, et al. Generativity among persons providing or receiving peer or mutual support: A scoping review. Psychiatric Rehabilitation Journal [Internet]. 2021 Oct 7 [cited 2021 Nov 30]; Available from: http://doi.apa.org/getdoi.cfm?doi=10.1037/prj0000498

6. Liang D, Jia R, Zhou X, Lu G, Wu Z, Yu J, et al. The effectiveness of peer support on self-efficacy and self-management in people with type 2 diabetes: A meta-analysis. Patient Educ Couns. 2021 Apr;104(4):760–9.

7. Hossain SN, Jaglal SB, Shepherd J, Perrier L, Tomasone JR, Sweet SN, et al. Web-Based Peer Support Interventions for Adults Living With Chronic Conditions: Scoping Review. JMIR Rehabil Assist Technol. 2021 May 25;8(2):e14321.

8. Shalaby RAH, Agyapong VIO. Peer Support in Mental Health: Literature Review. JMIR Ment Health. 2020 Jun 9;7(6):e15572.

9. Solomon P. Peer Support/Peer Provided Services Underlying Processes, Benefits, and Critical Ingredients. Psychiatric Rehabilitation Journal. 2004;27(4):392–401.

10. Berg RC, Page S, Øgård-Repål A. The effectiveness of peer-support for people living with HIV: A systematic review and meta-analysis. PLoS One. 2021;16(6):e0252623.

11. Kun KE, Couto A, Jobarteh K, Zulliger R, Pedro E, Malimane I, et al. Mozambique's Community Antiretroviral Therapy Support Group Program: The Role of Social Relationships in Facilitating HIV/AIDS Treatment Retention. AIDS Behav. 2019 Sep;23(9):2477–85.

12. McLeish J, Redshaw M. Mothers' accounts of the impact on emotional wellbeing of organised peer support in pregnancy and early parenthood: a qualitative study. BMC Pregnancy Childbirth. 2017 Jan 13;17(1):28.

13. Gruß I, Firemark A, Davidson A. Motherhood, substance use and peer support: Benefits of an integrated group program for pregnant and postpartum women. J Subst Abuse Treat. 2021 Dec;131:108450.

14. O'Mara-Eves A, Brunton G, Oliver S, Kavanagh J, Jamal F, Thomas J. The effectiveness of community engagement in public health interventions for disadvantaged groups: a meta-analysis. BMC Public Health. 2015 Feb 12;15(1):129.

15. Lauckner HM, Hutchinson SL. Peer support for people with chronic conditions in rural areas: a scoping review. Rural Remote Health. 2016 Mar;16(1):3601.

16. Maxwell C, Aggleton P, Warwick I. Involving HIV-positive people in policy and service development: Recent experiences in England. null. 2008 Jan 1;20(1):72–9.

17. Carter A, Greene S, Nicholson V, O'Brien N, Sanchez M, de Pokomandy A, et al. Breaking the Glass Ceiling: Increasing the Meaningful Involvement of Women Living With HIV/AIDS (MIWA) in the Design and Delivery of HIV/AIDS Services. null. 2015 Aug 3;36(8):936–64.

18. Arksey H, O'Malley L. Scoping studies: towards a methodological framework. null. 2005 Feb 1;8(1):19–32.

19. Levac D, Colquhoun H, O'Brien KK. Scoping studies: advancing the methodology. Implementation Science. 2010 Sep 20;5(1):69.

20. Veritas Health Innovation. Covidence systematic review software [Internet]. Melbourne, Australia; Available from: www.covidence.org

21. Backhaus S, Ibarra S, Parrott D, Malec J. Comparison of a Cognitive-Behavioral Coping Skills Group to a Peer Support Group in a Brain Injury Population. Arch Phys Med Rehabil. 2016 Feb;97(2):281–91.

22. Badger K, Royse D. Helping others heal: burn survivors and peer support. Social work in health care. 2010;49(1):1–18.

23. MacEachen E, Kosny A, Ferrier S. Unexpected barriers in return to work: lessons learned from injured worker peer support groups. Work. 2007;29(2):155–64.

24. Jalovcic D, Pentland W. Accessing Peers' and Health Care Experts' Wisdom: A Telephone Peer Support Program for Women with SCI Living in Rural and Remote Areas. Topics in Spinal Cord Injury Rehabilitation. 2009 Aug 16;15(1):59–74.

25. Stewart R, Bhagwanjee A. Promoting group empowerment and self-reliance through participatory research: a case study of people with physical disability. Disabil Rehabil. 1999 Jul;21(7):338–45.

26. Schulz CH. Helping factors in a peer-developed support group for persons with head injury, Part 2: Survivor interview perspective. Am J Occup Ther. 1994 Apr;48(4):305–9.

27. Morris M, Daluiski A, Dy C. A Thematic Analysis of Online Discussion Boards for Brachial Plexus Injury. The Journal of hand surgery [Internet]. 2016 Aug [cited 2023 Mar 9];41(8). Available from: https://pubmed.ncbi.nlm.nih.gov/27311862/

28. Schwartzberg SL. Helping factors in a peer-developed support group for persons with head injury, Part 1: Participant observer perspective. Am J Occup Ther. 1994 Apr;48(4):297–304.

29. Mignone J, Guidotti TL. Support groups for injured workers: process and outcomes. J Occup Environ Med. 1999 Dec;41(12):1059–64.

30. Williams MH. The self-help movement in head injury. Rehabil Nurs. 1990;15(6):311–5.

31. Whiting JB, Davies BN, Eisert BC, Witting AB, Anderson SR. Online Conversations About Abuse: Responses to IPV Survivors from Support Communities. J Fam Violence. 2022 Jul 22;1–11.

32. Levy BB, Luong D, Bayley MT, Sweet SN, Voth J, Kastner M, et al. A Pilot Feasibility Randomized Controlled Trial on the Ontario Brain Injury Association Peer Support Program. J Clin Med. 2021 Jun 29;10(13):2913.

33. Lau SKC, Luong D, Sweet SN, Bayley M, Levy BB, Kastner M, et al. Using an integrated knowledge translation approach to inform a pilot feasibility randomized controlled trial on peer support for individuals with traumatic brain injury: A qualitative descriptive study. PLoS One. 2021;16(8):e0256650.

34. Gelech J, Bayly M, Desjardins M. Constructing robust selves after brain injury: positive identity work among members of a female self-help group. Neuropsychol Rehabil. 2019 Apr;29(3):456–76.

35. Badger K, Royse D. Adult burn survivors' views of peer support: a qualitative study. Soc Work Health Care. 2010;49(4):299–313.

36. Grieve B, Shapiro GD, Wibbenmeyer L, Acton A, Lee A, Marino M, et al. Long-Term Social Reintegration Outcomes for Burn Survivors With and Without Peer Support Attendance: A Life Impact Burn Recovery Evaluation (LIBRE) Study. Arch Phys Med Rehabil. 2020 Jan;101(1S):S92–8.

37. Davis T, Gorgens K, Shriberg J, Godleski M, Meyer L. Making meaning in a burn peer support group: qualitative analysis of attendee interviews. J Burn Care Res. 2014;35(5):416–25.

38. Kira IA, Ahmed A, Wasim F, Mahmoud V, Colrain J, Rai D. Group therapy for refugees and torture survivors: treatment model innovations. Int J Group Psychother. 2012 Jan;62(1):69–88.

39. Scanlon-Schilpp AM, Levesque J. Helping the patient cope with the sequelae of trauma through the self-help group approach. J Trauma. 1981 Feb;21(2):135–9.

40. Molina O, Lawrence SA, Azhar-Miller A, Rivera M. Divorcing Abused Latina Immigrant Women's Experiences With Domestic Violence Support Groups. Journal of Divorce & Remarriage. 2009 Sep 30;50(7):459–71.

41. Morales-Campos DY, Casillas M, McCurdy SA. From isolation to connection: understanding a support group for Hispanic women living with gender-based violence in Houston, Texas. J Immigr Minor Health. 2009 Feb;11(1):57–65.

42. Balmer BR, Sippola J, Beehler S. Processes and outcomes of a communalization of trauma approach: Vets & Friends community-based support groups. J Community Psychol. 2021 Sep;49(7):2764–80.

43. Wolf RS. Support groups for older victims of domestic violence. J Women Aging. 2001;13(4):71–83.

44. Larance L, Porter M. Observations From Practice: Support Group Membership as a Process of Social Capital Formation Among Female Survivors of Domestic Violence | Office of Justice Programs. Journal of Interpersonal Violence. 2004 Jun;19(6):676–90.

45. Watson L, Andrews L. The effect of a Trauma Risk Management (TRiM) program on stigma and barriers to help-seeking in the police. International Journal of Stress Management. 2018;25:348–56.

46. Rivara F, Adhia A, Lyons V, Massey A, Mills B, Morgan E, et al. The Effects Of Violence On Health. Health Affairs. 2019 Oct;38(10):1622–9.

47. Craig A, Tran Y, Guest R, Gopinath B, Jagnoor J, Bryant RA, et al. Psychological impact of injuries sustained in motor vehicle crashes: systematic review and meta-analysis. BMJ Open. 2016 Sep 1;6(9):e011993.

48. Hardy BM, King KL, Enninghorst N, Balogh ZJ. Trends in polytrauma incidence among major trauma admissions. Eur J Trauma Emerg Surg [Internet]. 2022 Dec 19 [cited 2023 Jul 8]; Available from: https://doi.org/10.1007/s00068-022-02200-w

49. Carr MJ, Badiee J, Benham DA, Diaz JA, Calvo RY, Sise CB, et al. Fragmentation of care in the blunt abdominal trauma patient: Capturing our true outcomes and impact on care. J Trauma Acute Care Surg. 2021 Nov 1;91(5):829–33.

50. Nantulya VM, Reich MR. The neglected epidemic: road traffic injuries in developing countries. BMJ. 2002 May 11;324(7346):1139–41.

51. Global Burden of Disease 2016 Injury Collaborators, Naghavi M, Marczak LB, Kutz M, Shackelford KA, Arora M, et al. Global Mortality From Firearms, 1990-2016. JAMA. 2018 Aug 28;320(8):792–814.

52. Rissanen R, Ifver J, Hasselberg M, Berg HY. Quality of life following road traffic injury: the impact of age and gender. Qual Life Res. 2020 Jun 1;29(6):1587–96.

53. Dain K. A new chapter for the NCD Alliance: stronger together. The Lancet. 2019 May 27;389(10084):2089–90.

54. Meara JG, Leather AJM, Hagander L, Alkire BC, Alonso N, Ameh EA, et al. Global Surgery 2030: evidence and solutions for achieving health, welfare, and economic development. The Lancet. 2015;386(9993):569–624.

55. Riddell MA, Edwards N, Thompson SR, Bernabe-Ortiz A, Praveen D, Johnson C, et al. Developing consensus measures for global programs: lessons from the Global Alliance for Chronic Diseases Hypertension research program. Global Health. 2017 Mar 15;13(1):17.

56. Brownson CA, Heisler M. The role of peer support in diabetes care and self-management. Patient. 2009 Mar 1;2(1):5–17.

57. Brooks H, Devereux-Fitzgerald A, Richmond L, Bee P, Lovell K, Caton N, et al. Assessing the effectiveness of social network interventions for adults with a diagnosis of mental health problems: a systematic review and narrative synthesis of impact. Soc Psychiatry Psychiatr Epidemiol. 2022 May;57(5):907–25.

58. Parry M, Watt-Watson J. Peer support intervention trials for individuals with heart disease: a systematic review. Eur J Cardiovasc Nurs. 2010 Mar;9(1):57–67.

59. Wasilewski MB, Rios J, Simpson R, Hitzig SL, Gotlib Conn L, MacKay C, et al. Peer support for traumatic injury survivors: a scoping review. Disabil Rehabil. 2022 Jun 9;1–34.

60. Shanthakumar D, Payne A, Leitch T, Alfa-Wali M. Trauma Care in Low- and Middle-Income Countries. Surg J (N Y). 2021 Oct 22;7(4):e281–5.

61. Fisher EB. Global Evidence for Peer Support: Humanizing Health Care [Internet]. Peers for Progress; National Council of La Raza; 2014 [cited 2023 Mar 21]. Available from: https://www.ipfcc.org/bestpractices/global-evidence-for-peer-support.pdf

62. Øgård-Repål A, Berg RC, Fossum M. Peer Support for People Living With HIV: A Scoping Review. Health Promot Pract. 2023 Jan;24(1):172–90.

63. Ayala G, Sprague L, Merwe LLA van der, Thomas RM, Chang J, Arreola S, et al. Peer- and community-led responses to HIV: A scoping review. PLOS ONE. 2021 Dec 1;16(12):e0260555.

64. Park HY, Kim MJ, Kim JY, Kim S, Choi JY, Kim JH, et al. Could Peer Support Programs Be a Good Resource for Managing the Unmet Needs of Cancer Patients? J Canc Educ. 2019 Oct 1;34(5):950–7.

65. Lyons N, Cooper C, Lloyd-Evans B. A systematic review and meta-analysis of group peer support interventions for people experiencing mental health conditions. BMC Psychiatry. 2021 Jun 23;21(1):315.

66. Arjadi R, Nauta MH, Chowdhary N, Bockting CLH. A systematic review of online interventions for mental health in low and middle income countries: a neglected field. Glob Ment Health (Camb). 2015;2:e12.

SUPPLEMENT 1: SEARCH STRATEGIES BY DATABASE

PubMed / Medline (National Library of Medicine, NCBI)
2,343 records March 1, 2023

("Self-Help Groups"[Mesh:NoExp] OR community engagement[tiab] OR community group*[tiab] OR community support[tiab] OR help group*[tiab] OR patient support[tiab] OR peer support[tiab] OR peer counsel*[tiab] OR support group*[tiab] OR online support[tiab])

AND
("Wounds and Injuries"[Mesh] OR "Accidents"[Mesh] OR "Violence"[Mesh] OR accidental[tiab] OR accidents[tiab] OR burn[tiab] OR burns[tiab] OR domestic abuse[tiab] OR fallers[tiab] OR falls[tiab] OR fractures[tiab] OR gunshot[tiab] OR injuries[tiab] OR injury[tiab] OR injured[tiab] OR partner abuse[tiab] OR physical abuse[tiab] OR spousal abuse[tiab] OR spouse abuse[tiab] OR stabbing*[tiab] OR trauma[tiab] OR traumas[tiab] OR wound[tiab] OR wounds[tiab] OR violence[tiab])

Cumulative Index to Nursing and Allied Health Literature (CINAHL Complete, EBSCOhost)
1077 records, March 1, 2023

Advanced Search

Search Options
remove:
Apply equivalent subjects

select:
Publication Type: Journal Article

MH "Support Groups" OR MH "Peer Counseling"

OR title OR abstract:

"community engagement" OR "community group*" OR "community support" OR "help group*" OR "patient support" OR "peer support" OR "support group*" OR "online support"

AND

MH "Wounds and Injuries+" OR MH "Accidents+" OR MH "Trauma" OR MH "Violence+"

OR title OR abstract

accidental OR accidents OR burn OR burns OR "domestic abuse" OR fallers OR falls OR fractures OR gunshot OR injuries OR injury OR injured OR "partner abuse" OR "physical abuse" OR "spousal abuse" OR "spouse abuse" OR stabbing* OR trauma OR traumas OR wound OR wounds OR violence

Web of Science Core Collection (Clarivate)

1,569 records, March 1, 2023

Editions:
Science Citation Index Expanded
Social Sciences Citation Index

Advanced Search
More Options: Exact Search

#1
(TI=("community engagement" OR "community group*" OR "community support" OR "help group*" OR "patient support" OR "peer support" OR "support group*" OR "online support")) OR AB=("community engagement" OR "community group*" OR "community support" OR "help group*" OR "patient support" OR "peer support" OR "support group*" OR "online support")

AND

(TI=(accidental OR accidents OR burn OR burns OR domestic abuse OR fallers OR falls OR fractures OR gunshot OR injuries OR injury OR injured OR "partner abuse" OR "physical abuse" OR "spousal abuse" OR "spouse abuse" OR stabbing* OR trauma OR traumas OR wound OR wounds OR violence)) OR (AB=(accidental OR accidents OR burn OR burns OR domestic abuse OR fallers OR falls OR fractures OR gunshot OR injuries OR injury OR injured OR "partner abuse" OR "physical abuse" OR "spousal abuse" OR "spouse abuse" OR stabbing* OR trauma OR traumas OR wound OR wounds OR violence))

#1 AND #2

PubMed: 2,343
CINAHL: 1077
Web of Science Core Collection: 1,569

total # of references retrieved across 3 databases March 1, 2023: 4,989

PONE-D-23-23344
Community Support for Injured Patients: A Scoping Review and Narrative Synthesis
Response to Reviewers

Dear Reviewers and Academic Editor,

Thank you very much for your thorough evaluation of our manuscript, “Community Support for Injured Patients: A Scoping Review and Narrative Synthesis.” We have responded to the comments and suggestions made by all reviewers, and edited the manuscript as suggested. Please see below for our point-by-point responses. Both a marked/track changes version and unmarked version of the revised manuscript have also been submitted for review.

Best,
Rashi Jhunjhunwala and co-authors.

Reviewer #1

**Comment**	**Rebuttal**
Although the topic of study is very important, the article is not suitable for publication.	We hope you will change this opinion after reviewing our revised manuscript.
Range of time interval of studies under review is very wide.	We do not view this as a detriment to our study. We aimed to evaluate the research that has been done on community-based peer support groups for injured patients, and we wanted to include all research that we were able to access. We did not see a need to limit our search to any specific time frame.
Table 1 could have been presented in prose, Table 2 should have been attached to the article.	We chose to present table 1 in table format because we found it easier to quickly reference inclusion/exclusion criteria in this manner. Additionally, this is a format that is commonly used in scoping reviews. Table 2 is included at the bottom of the manuscript and was able to be located by other reviewers.
Poor graphs are presented, no statistical methods are used to answer the researcher's question, and it does not add new findings to the existing literature.	The methodology utilized in this review is detailed in the Methods section of the manuscript. While no statistical methods were included, there are other valid research methodologies that exist; one of these is narrative synthesis. We refer the reviewer to the following publication from the Joanna Briggs Institute to gain insight into the ways data can be analyzed and presented in a scoping review: Pollock, Danielle1; Peters, Micah D.J.2,3,4; Khalil, Hanan5; McInerney, Patricia6; Alexander, Lyndsay7,8; Tricco, Andrea C.9,10,11; Evans, Catrin12; de Moraes, Érica Brandão13,14; Godfrey, Christina M.11; Pieper, Dawid15,16; Saran, Ashrita17,18; Stern, Cindy1; Munn, Zachary1. Recommendations for the extraction, analysis, and presentation of results in scoping reviews. JBI Evidence Synthesis 21(3):p 520-532, March 2023. | DOI: 10.11124/JBIES-22-00123

Reviewer #2

Comment	Rebuttal
The studies in this review identified positive benefits of community support groups for injured people, including creation of a group identity and regaining of self-esteem, emotional support and guidance, and delivery of practical knowledge about their disease, recovery and treatment process, or ways to access medical, legal, or social support. However, there are inconsistencies in the fonts, some of which are neo-Roman. Please correct it as requested by PLOS ONE.	Fonts have been standardized to adhere to PLOS ONE guidelines.

Reviewer #3

Comment	Rebuttal
The aim of the review appears broader than what is initially stated in the research question. Clarifying the alignment between the research question and the review's objectives would enhance the paper's coherence.	Thank you for this comment. We have revised the aims and research question.<br><br>The aims now read: “This scoping review aims to identify the extent, distribution, benefits, utility, and impact of community-based peer support groups for injured patients”.<br><br>The research question reads: “what research has been done on the extent, distribution, benefits, utility, and impact of health-system independent community-based support groups (i.e. those which are not based in healthcare facilities or run by healthcare professionals) for injured persons?”
It's noted that 13 studies were excluded due to unavailability of full texts, but there is no mention of efforts made to retrieve them, such as contacting the authors. Additionally, the potential impact of excluding these studies on the conclusions should be addressed in the limitations section.	This is a very valid point. Many of the results that we were unable to find were find were from the 1980s and early 1990s and contact information for the authors were not available. We have given more detail regarding the extent to which attempts were made to retrieve the articles in the first section of the “Results” – lines 180-183. The text now reads:<br><br>“Full text was not recoverable for 13 studies due to unavailability online through Harvard libraries, inter-library loan, or other formal retrieval mechanisms. Four of 13 unavailable studies were published prior to 1990 and contact information for the authors was not available. Attempts to contact the other 9 authors were unsuccessful.”<br><br>We have also included the fact that these were not retrieved in the limitations, as suggested (lines 448-449): There were 13 (out of 112) articles not

	found by our search strategy that could have added additional insight.
The discussion section requires a complete rewrite rather than just improvements. It lacks a clear flow and connection with the results section. Instead, it introduces new information that should have been integrated into the results. Furthermore, the emphasis on the scarcity of studies from low and middle-income countries diverts attention from discussing the actual results and comparing them with other studies.	The discussion has been re-structured to first state main results, discussion of those results, the context in the literature with other conditions, the unique challenges of injury/trauma in formthe lack of literature in LMICs, limitations, and conclusion. The discussion on LMICs and socioeconomic element has been paired down to discuss it as an important context to injury burden, but not to take away from the main results.
I have attached a file with more detailed comments for your reference.	Thank you for your thorough and thoughtful comments. We have aimed to respond to them in the track-changes document we have attached for review. These include:<br><br>- We have standardized the language regarding “community-based peer support groups” throughout the document, and included an acronym, CBPR.<br>- Revised aims and research question, as stated above.<br>- Included more information about missing studies, as stated above.<br>- Included references for each component of the “study characteristics” section of the Results.<br>- Defined each category of “social, emotional, educational, logistical” benefits immediately prior to “figure 2”.<br>- Revised/rewrote discussion, as stated above.<br>- Various sentences/wording have been revised as recommended.
