# Supplementary material for: Community support for injured patients: A scoping review and narrative synthesis
Source: PLoS One. 2024 Feb 1;19(2):e0289861. doi: 10.1371/journal.pone.0289861 (PMC10833531; doi:10.1371/journal.pone.0289861)
Supplement: Attachment — Submitted filename: Community Support Response to Reviewers 12.14.23.docx [file pone.0289861.s006.docx]

PONE-D-23-23344

Community Support for Injured Patients: A Scoping Review and Narrative Synthesis

Response to Reviewers

Dear Reviewers and Academic Editor,

Thank you very much for your thorough evaluation of our manuscript, “Community Support for Injured Patients: A Scoping Review and Narrative Synthesis.” We have responded to the comments and suggestions and edited the manuscript as suggested. Please see below for our point-by-point responses. Both a marked/track changes version and unmarked version of the revised manuscript have also been submitted for review.

Best,

Rashi Jhunjhunwala and co-authors.

**Reviewer #3**

| **Comment** | **Rebuttal** |
| --- | --- |
| Spacing (line 43) | Extra space has been removed |
| I previously commented on this issue. You need to decide whether the reference should be before or after the dot | We have standardized all references to be placed before the period. |
| Add a sentence about heterogeneity and how it could affect your findings (limitations paragraph) | The following sentence was added to the Limitations paragraph: “Furthermore, the studies included in this review were heterogeneous both in peer support group characteristics and study design, which limits the generalizability of these findings.” |
